# Multisite binding of bacteriophages on lipopolysaccharides in *Escherichia coli* O157:H7 and the adaptive costs of phage resistance

**DOI:** 10.1128/spectrum.00067-25

**Published:** 2025-06-17

**Authors:** Yang Wang, Jing Li, Yuhan Zhang, Yao Li, Xinru Chen, Jiaqi Cui, Feng Xue, Jianluan Ren, Jianjun Dai, Fang Tang

**Affiliations:** 1 Key Laboratory of Animal Bacteriology, Ministry of Agriculture, College of Veterinary Medicine,Nanjing Agricultural University 70578, , Nanjing, China; 2 School of Life Science and Technology, China Pharmaceutical University 56651, Nanjing, China; Universidad Nacional Autonoma de Mexico-Campus Morelos, Cuernavaca, Mexico

**Keywords:** *E. coli *O157:H7, phage, receptor, phage resistance, adaptive cost

## Abstract

*Escherichia coli* (*E. coli*) O157:H7 is a major foodborne pathogen and a critical global food safety concern. Bacteriophage (phage) therapy offers a promising, highly specific biological alternative to antibiotic treatment. However, the clinical application of phage therapy is frequently limited by the rapid emergence of bacterial resistance. In this study, we examined the interaction between *E. coli* O157:H7 strain EDL933 and two T4-like phages, assessing the adaptive costs incurred by host strains in developing phage resistance. Our results indicate that phage PSD2001 utilizes capsular polysaccharide and lipopolysaccharides as adsorption receptors, while phage PNJ212 targets outer membrane protein C (OmpC) as its adsorption receptor. Notably, both phage employ gp37-like protein as receptor-binding proteins, highlighting the diversity of receptors on EDL933 and the complexity of phage-host recognition mechanisms. Furthermore, this study identified the adaptive costs of phage resistance, including antibiotic susceptibility, biofilm formation ability, survival, and colonization abilities, in the environment. These findings deepen our understanding of phage-host interactions and offer valuable insights for the application of phage therapy.

**IMPORTANCE:**

Phage therapy offers an innovative strategy to combat antibiotic-resistant bacterial infections. To address the challenge of phage-resistant strains, we can adopt two strategies: using phage cocktails targeting multiple bacterial receptors to delay resistance development; and implementing a 'phage shift' treatment strategy that exploits the adaptive trade-offs of phage-resistant bacteria. Our research provides insights into the phage receptor recognition mechanisms in *Escherichia coli* O157:H7, a major foodborne pathogen. We identified key target receptors, including bacterial capsular polysaccharide, lipopolysaccharides, and OmpC, and found that the receptor-binding strategies of these phages resemble those of the T4 phage tail fiber protein gp37. Additionally, we revealed the adaptive costs associated with bacterial resistance to phage, which can inform strategies to enhance phage therapy efficacy. In summary, our findings provide a theoretical foundation for the prevention and control of clinical *E. coli* O157:H7 strains.

## INTRODUCTION

The hemorrhagic *Escherichia coli* serotype O157:H7 was first identified in 1982 in cases of hemorrhagic diarrhea in Oregon and Michigan, USA (1, 2) and has since become a significant threat to food safety and public health. In 1983, this serotype was linked to sporadic cases of hemolytic uremic syndrome (HUS) (3). *E. coli* O157:H7 can adhere to intestinal epithelial cells and produce Shiga toxin, leading to severe diseases, such as hemorrhagic colitis, HUS, and thrombotic thrombocytopenic purpura (4). Amid rising antibiotic resistance, traditional antibiotic treatment strategies are increasingly challenged by the emergence of multidrug-resistant, pan-resistant, and even ’superbug' strains that are resistant to all antibiotics, thereby diminishing their effectiveness (5).

In this context, phage therapy has re-emerged as an alternative or complementary therapeutic strategy (6). Phages, among the most abundant biological entities on Earth, play a critical ecological role in the dynamics, activity, and adaptability of microbial communities (7). However, phage therapy also faces the challenge of bacterial resistance (8). Mechanisms of bacterial resistance to phage include blocking phage adsorption (9), preventing phage DNA penetration (10), restricting modification systems (11), and disrupting infection systems (12, 13). To enhance the antimicrobial efficacy of phage, it is crucial to understand phage-host interactions and concurrently investigate the fitness costs incurred by hosts in response to phage pressure (14).

The first step in phage infection is the specific adsorption of the phage to host surface receptors often likened to the interaction between a lock (host receptor) and a key (RBP) (15). Bacteria evade phage infection through genetic mutations that disrupt or impair the synthesis of adsorption receptors. Phage receptors are typically surface components of bacteria, including lipopolysaccharides (LPS), flagella, fimbriae, and capsular polysaccharide (CPS). *E. coli* synthesizes capsules via four distinct mechanisms classified into groups I to IV (16). Group IV capsules, also known as O-antigen capsules (17), are encoded by the gfc operon, which includes seven genes: *cspH*, *ymcD*, *ymcC*, *ymcD*, *ymcA*, *ymcZ*, *etp*, *etk*, and *apaA* (Fig. S1A). The proteins encoded by these genes are essential for the repeated polymerization of polysaccharides into the O-antigen capsule. *E. coli* O157:H7 forms O antigen capsule, but it remains unclear whether CPS serves as a receptor for *E. coli* O157:H7 phage. LPS is a common phage receptor, and in *E. coli*, the LPS core region is classified into five distinct types—K-12, R1, R2, R3, and R4—based on their sugar composition. Specifically, *E. coli* B is classified as R1 type, *E. coli* K-12 as K-12 type, and O157:H7 as R3 type (18). In the R3 type, the main structure of the external polysaccharide consists of four monosaccharides and one glucosamine. The sugar composition is arranged as glucose (Glc) I, galactose (Gal) I, Glc II, and Glc III, from the inside to the outside, with Gal I linked to two glucose units at both ends and an N-acetylglucosamine (GlcNAc) at one end (Fig. S1B). The genes responsible for synthesizing glycosyltransferases include *waaG*, *waaO*, *waaR*, and *waaD*, while the *waaL* gene encodes the enzyme that transfers the O antigen to Glc II (Fig. S1B). Currently, the classic T4 phage is known to use the LPS of *E. coli* B as its receptor, with key adsorption sites on Glc I and Glc II (18). Qianwen et al. determined that LPS serves as secondary receptors of phage PNJ1809-36 to *E. coli* DE058 and pinpointed the penultimate galactose in the outer core as the specific binding site on LPS (19). However, the specific binding sites of phage on the R3-type LPS of *E. coli* O157:H7 remain unclear. The RBP of phage is a protein responsible for binding to receptors on the bacterial surface. The structure of RBPs consists of a conserved N-terminal domain, a variable hinge region, and a C-terminal domain, which can adsorb and hydrolyze bacterial surface receptors (20). For example, the gp37 protein in the long-tailed filament of phage T4 serves as a receptor-binding domain and can reversibly bind to LPS or OmpC on the surface of *E. coli* (21). The short-tailed phage (Podoviridae) P22 recognizes bacterial outer membrane LPS via the C-terminus of its tail spike protein gp9 (22). Bacteria acquire phage resistance through receptor modulation, but this process may incur a fitness cost. Combined treatment with phages and subinhibitory concentrations of antibiotics has been shown to effectively eliminate resistant mutants and enhance therapeutic efficacy (23, 24). Additionally, phage-resistant strains may exhibit increased susceptibility to antibiotics, providing a theoretical basis for combined phage-antibiotic treatment.

**Fig 1:**
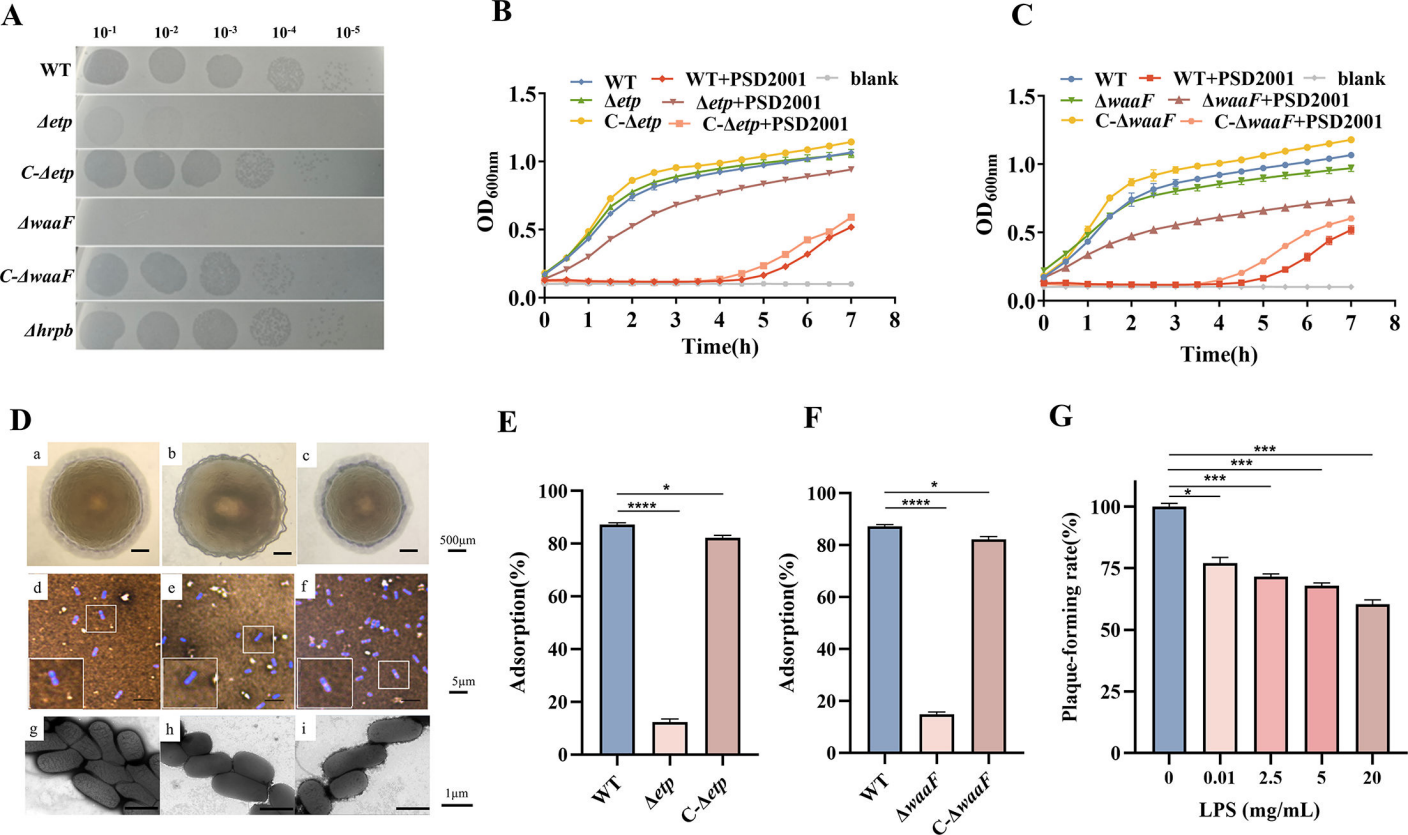
CPS and LPS as adsorption receptors for phage PSD2001. (A) Spot assays. The spot assays were conducted by applying continuously diluted phage solutions onto plates inoculated with different bacterial strains. (B) Lysis curves of EDL933, Δ*etp*, and C-Δ*etp* strains. (C) Lysis curves of WT, Δ*waaF*, and C-Δ*waaF* strains. (D) Capsule morphology. Colony morphology of WT (a), Δ*etp* (b), and C-Δ*etp* (C); capsule staining of WT (D), Δ*etp* (E), and C-Δ*etp* (F); electron micrographs of WT (G), Δ*etp* (H), and C-Δ*etp* (I). Scale bars: 500 µm (a to c), 5 µm (d to f), and 1 µm (g to i). (E) The number of free phage was measured after adsorption to WT, Δ*etp*, and C-Δ*etp* strains for 10 min. All assays were performed in triplicate. (F) The number of free phage was measured after adsorption to WT, Δ*waaF*, and C-Δ*waaF* for 10 min. (G) Different concentrations of LPS were added to compete for LPS receptor *in vitro*. Data are presented as means ± standard deviations from triplicate experiments. *, *P* < 0.05; ***, *P* < 0.001; ****, *P* < 0.0001.

In this study, two T4-like phages, PSD2001 and PNJ212, each possessing a distinct host range yet both specifically targeting *E. coli* O157:H7 strain EDL933, were selected. By screening resistant strains arising from natural mutations and performing genomic analysis of these mutants using next-generation sequencing (NGS), we identified gene mutations associated with phage resistance and characterized the adsorption receptors and corresponding RBPs for both phages. By assessing the performance of these resistant strains in terms of antibiotic sensitivity, biofilm formation ability, and survival in serum, we further investigated the adaptive costs of phage resistance. These results enhance our understanding of phage-host recognition mechanisms and provide a molecular basis for optimizing phage therapy.

## RESULTS

### Mutations in the *waaF* and *etp* genes confer resistance to phage PSD2001

The phage PSD2001 belongs to the Myoviridae family and shares 97.13% homology with T4 phage P479 (accession number MW269952.1). PSD2001 and *E. coli* EDL933 were spread using the double-layer agar method and incubated at 37°C for 24 h. Single colonies emerging from phage plaques were considered potential resistant candidates. A total of 50 phage-resistant colonies were selected and numbered from SD0101 to SD0150. In the absence of phage pressure, most of these phage-resistant strains lost their phage resistance over successive passages. However, SD0102 and SD0123 remained stably resistant to phage after passage. Double-layer agar plate assays of SD0102 and SD0123 did not produce distinct plaques when exposed to 10⁴ PFU/mL of PSD2001 (Fig. S2). DNA from SD0102 and SD0123 was extracted for NGS, and assembly analysis revealed that SD0102 harbors a 22 bp deletion in the *waaF* gene, which is involved in LPS synthesis. In contrast, SD0123 exhibited SNP mutations in the *etp* gene associated with O-antigen capsule synthesis and the *hrpB* gene, which encodes an ATP-dependent helicase (Table 1). To verify the association between the three mutant genes (*etp*, *waaF*, and *hrpB*) and phage resistance, we constructed deletion strains (Δ*etp*, Δ*waaF*, Δ*hrpB*) and plasmid complementation strains (C-Δ*etp*, C-Δ*waaF*). Phage spot tests and phage lysis curve assays were then performed using these strains. In the spot test, phage plaques were observed at high phage titers on strain Δ*etp* but were absent at low titers. Phage plaques did not form on strain Δ*waaF*, regardless of the phage titer and the complemented strains (C-Δ*etp*, C-Δ*waaF*) restored sensitivity to PSD2001 (Fig. 1A). However, the deletion of *hrpB* did not significantly affect phage lysis (Fig. 1A). The phage lysis curve revealed that the OD_600_ of Δ*etp* and Δ*waaF* strains continued to increase after the addition of PSD2001 compared to the wild-type (WT) strain (Fig. 1B and C). In contrast, the increasing trend of C-Δ*etp* and C-Δ*waaF* was partially restored (Fig. 1B and C). These results indicate that phage resistance is associated with mutations in the *etp* and *waaF* genes.

**Fig 2:**
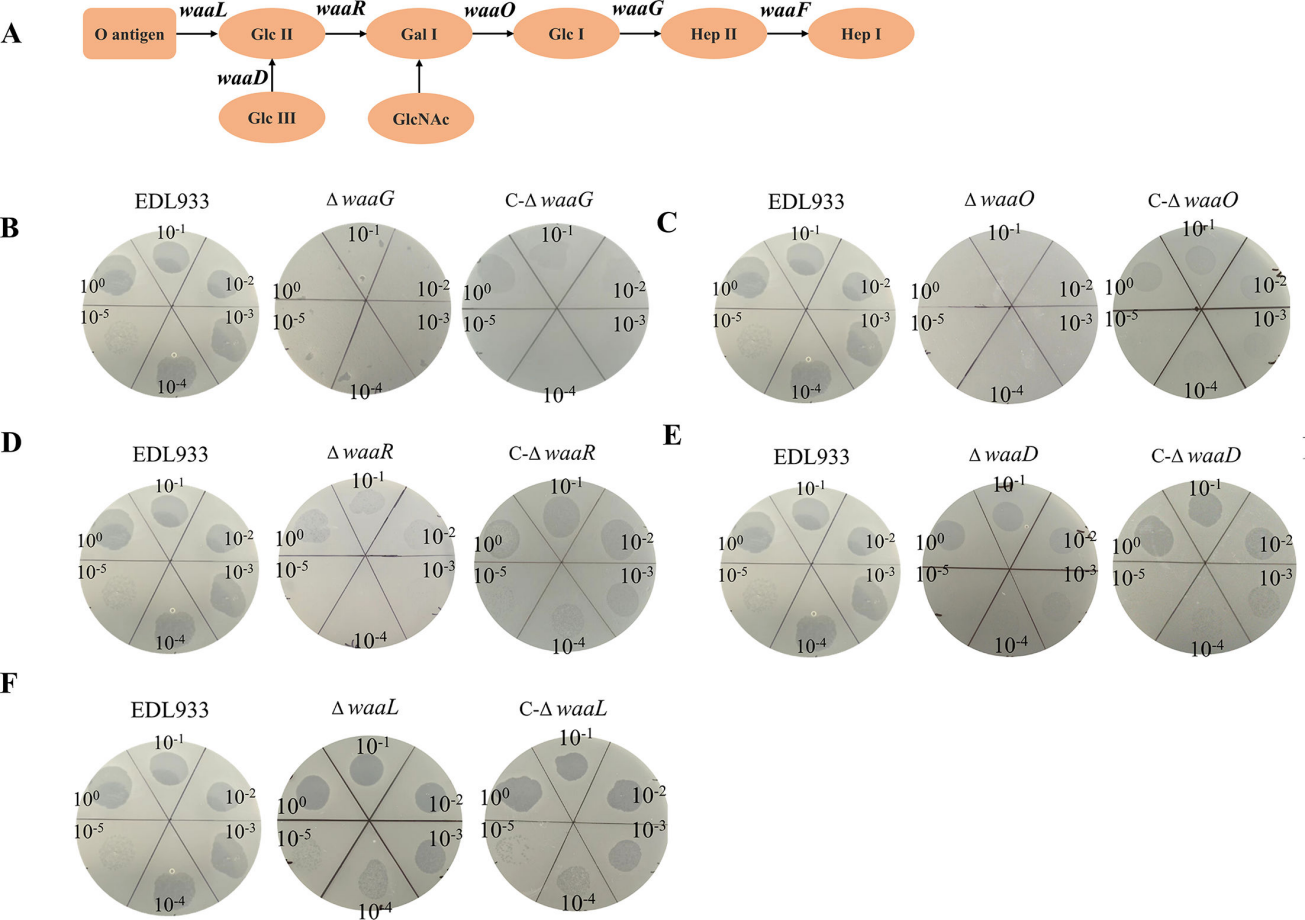
LPS as the secondary receptor. (A) LPS structure. (B) Spot assays in gene deletion mutant Δ*waaG.* (C) Spot assays in gene deletion mutant Δ*waaO.* (D) Spot assays in gene deletion mutant Δ*waaR.* (E) Spot assays in gene deletion mutant *ΔwaaD.* (F) Spot assays in gene deletion mutant *ΔwaaL.*

**TABLE 1:** Summary of identified phage resistance mutations

Resistant isolate	Mutation gene	Mutation	Putative gene function
SD0102	*waaF*	22 base deletions from 789 to 810 bp	LPS heptosyltransferase
SD0123	*etp*	A base G insertion from 119 to 120 bp	O-antigen capsule forming protein-tyrosine-phosphatase
SD0123	*hrpB*	Nine base deletions from 958 to 966 bp	ATP-dependent helicase

### The O-antigen capsule serves as the primary receptor for phage PSD2001 adsorption

EDL933 produces the O-antigen capsule, and inactivation of *etk* or *etp* results in loss of the O-antigen polysaccharide. The *etp* deletion mutant (Δ*etp*) and the complementary strain (C-Δ*etp*) were generated. Colonies of the WT strain (Fig. 1D–a) and C-Δ*etp* (Fig. 1D–c) exhibited smooth edges, whereas the Δ*etp* colony showed a rough edge (Fig. 1D–b). After crystal violet staining, the capsule is not stained, so transparent halos were observed on the surface of the WT strain (Fig. 1D–d) and C-Δ*etp* (Fig. 1D–f), indicating the presence of the capsule, whereas no halos were seen in Δ*etp* (Fig. 1D–e). Transmission electron microscopy revealed a thicker capsular polysaccharide on the cell surface of the WT strain (Fig. 1D–g) and C-Δ*etp* (Fig. 1D–i) compared to Δ*etp* (Fig. 1D–h). As shown in Fig. 1E, phage adsorption efficiency was reduced by nearly 80% in the Δ*etp* strain relative to the WT but was nearly restored to WT levels in the complement strain. The results indicate that the O-antigen capsule is the primary receptor for phage PSD2001 adsorption.

### LPS serves as a secondary receptor for phage PSD2001 binding, with specific binding sites identified as Glc II, Glc III, and Gal I in the outer core polysaccharide

The *waaF* gene encodes glycosyltransferases involved in LPS biosynthesis. To further investigate whether LPS serves as the phage receptor, we tested the adsorption efficiency of a *waaF* deletion strain. The results demonstrated that the phage PSD2001 exhibited a 60% reduction in adsorption efficiency on the Δ*waaF* strain compared to the WT. This phenotype was restored in the complemented strain (Fig. 1F). Furthermore, the addition of exogenous LPS inhibited phage adsorption to host bacteria in a concentration-dependent manner (Fig. 1G). These findings indicate that LPS is a critical receptor for phage PSD2001 infection of EDL933.

The *waaF* gene catalyzes the transfer of heptose to the inner nucleooligosaccharide of LPS (Fig. 2A). To identify the specific LPS binding sites for PSD2001, deletion and complementation strains of *waaG*, *waaO*, *waaR*, *waaD*, and *waaL* were constructed. Dot spot assay results revealed the absence of plaque formation on the Δ*waaG* (Fig. 2B) and Δ*waaO* strains (Fig. 2C), indicating that the phage binding site is located on structures external to the core outer polysaccharide Glc I. PSD2001 partially restored sensitivity on the Δ*waaR* strain (Fig. 2D), suggesting that Gal I serves as a binding site. Subsequently, compared to Δ*waaR*, the O antigen and Glc II were more exposed in Δ*waaD*, and plaque assays revealed that Δ*waaD* exhibited greater sensitivity than Δ*waaR* (Fig. 2E), suggesting that Glc II contributes to additional binding sites. Δ*waaL* exhibited greater exposure of Glc III compared to Δ*waaD*, and plaque assays showed that Δ*waaL* displayed even higher sensitivity than Δ*waaD* (Fig. 2F) and was comparable to that on the WT, suggesting that Glc III also serves as a binding site, while O antigen is not a specific site. The results of the lysis curve and adsorption rate assays were consistent with the spot assay findings. For the Δ*waaG* and Δ*waaO* strains, the growth curves in the presence of phage PSD2001 showed no significant difference compared to those without the phage, indicating that PSD2001 exhibits complete resistance to the deletion of *waaG* and *waaO*, with a marked decrease in adsorption rate relative to the wild-type strain. In the case of Δ*waaR*, phage addition resulted in partial growth inhibition, and the adsorption rate was higher compared to Δ*waaG* and Δ*waaO*. As for Δ*waaD* and Δ*waaL*, their growth was significantly inhibited upon phage addition, and while their adsorption rates were lower than that of the wild-type strain, they were notably higher than those of the other deletion mutants. (Fig. S3A through E and S4A). Based on the above results, it is preliminarily concluded that the adsorption sites of phage PSD2001 are located on Glc II, Glc III, and Gal I. To further investigate the involvement of protein receptors, the adsorption efficiency of phage PSD2001 was assessed following treatment of the bacteria with proteinase K or periodic acid. As shown in Fig. S4B, the adsorption efficiency of EDL933 by PSD2001 remained unaffected by proteinase K treatment but significantly decreased following periodic acid treatment, confirming that phage PSD2001 utilizes polysaccharides as its sole receptors.

**Fig 3:**
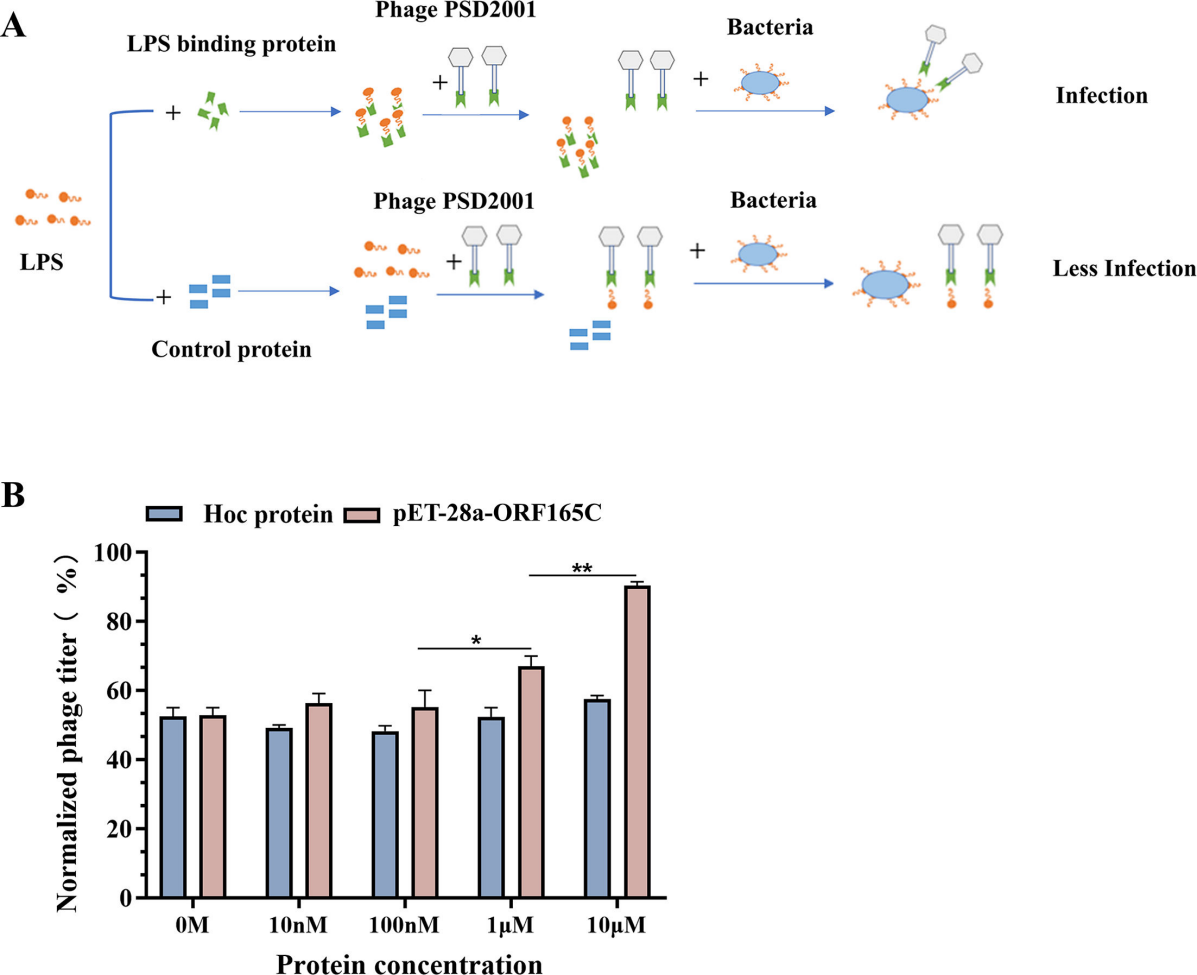
ORF165 as the RBP. (A) Schematic depicting the competition between RBP and phage for LPS binding. Exogenous LPS was pre-incubated with LPS-binding tail fiber protein or control protein, followed by the addition of phage PSD2001 for further incubation. The bacterial suspension was then introduced. In the experimental group, the exogenous LPS was bound by the expressed tail fiber protein, enabling the phage to adsorb onto bacterial LPS and initiate infection. In contrast, in the control group, the phage adsorbed to the exogenous LPS, resulting in reduced bacterial infection. (B) Phage titers measured following competition between varying concentrations of RBP and phage for LPS binding. *, *P* < 0.05; **, *P* < 0.01.

### Tail protein ORF165 is the RBP of phage PSD2001 binding to LPS

To identify the RBPs of phage PSD2001, bioinformatic analyses were conducted on all potential tail-associated proteins. The open reading frame (ORF)165 protein is predicted to be a distal long-tail protein, with its C-terminus showing 66.66% homology to the C-terminus of gp37 from phage BP7, which serves as the receptor-binding site for LPS of *E. coli* K12 (25). Therefore, we hypothesize that the ORF165 protein binds to LPS of EDL933 as an RBP. To test whether the C-terminally truncated ORF165 protein (pET-28a-ORF165C) interacts with LPS, a competitive inhibition assay was performed (Fig. 3A). Phage titers increased progressively with higher concentrations of the pET-28a-ORF165C (Fig. 3B). However, this increase was not observed at protein concentrations below 100 nM. As a negative control, no significant change in phage titers was detected following incubation of the Hoc protein with LPS. These results suggest that the tail protein ORF165 of PSD2001 is the RBP for host LPS, with the C-terminus serving as the receptor-binding site.

### The outer membrane protein OmpC acts as an additional receptor for another phage PNJ212

**Fig 4:**
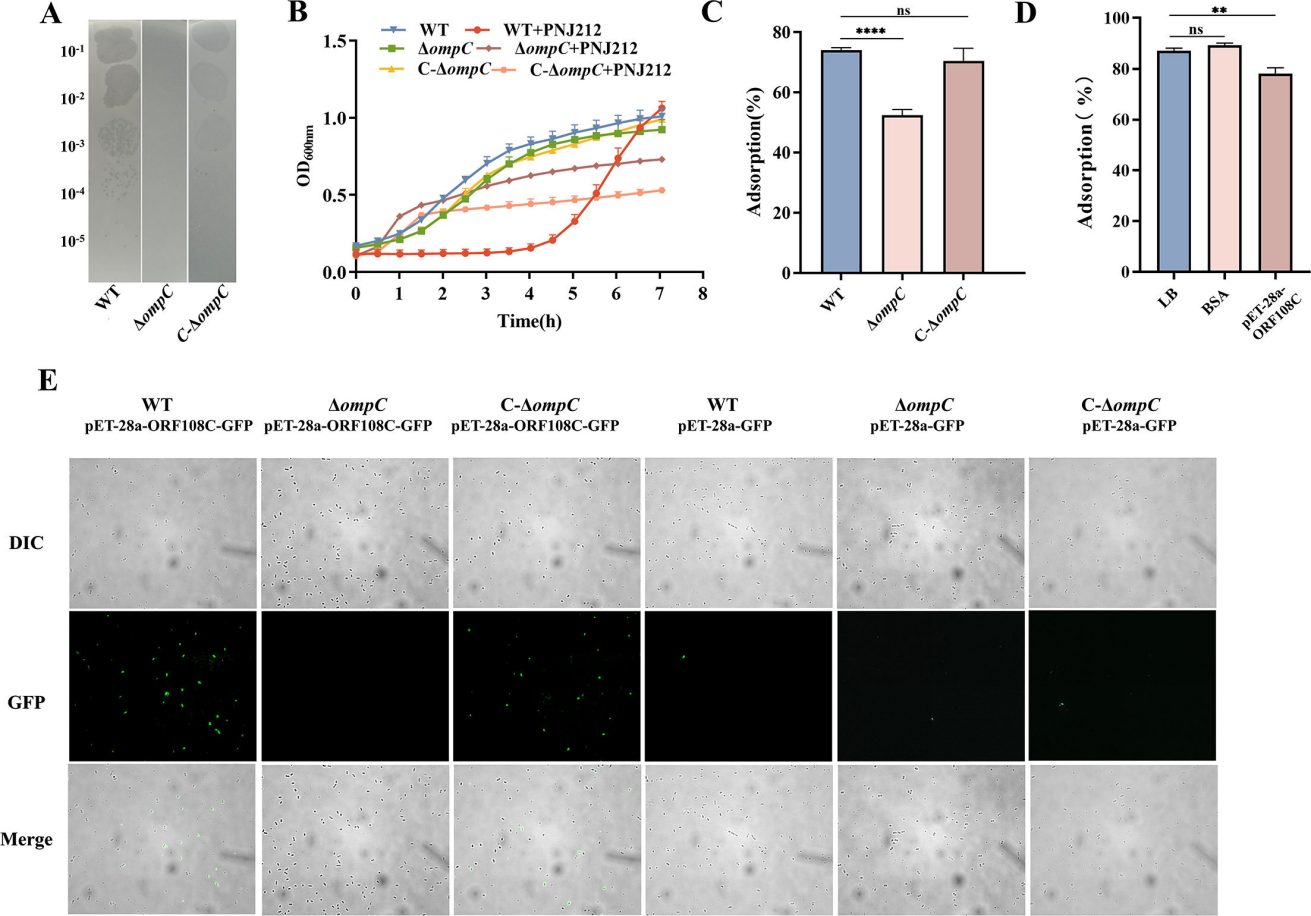
OmpC as the receptor for PNJ212 and ORF108 as the RBP. (A) Spot assays. (B )Lysis curves for WT, Δ*ompC*, and C-Δ*ompC*. (C) Number of free phage detected after 10 min of adsorption to WT, Δ*ompC*, and C-Δ*ompC*. (D) Determination of the adsorption rate of phage PNJ212 to WT with or without pre-incubation with the C-terminally truncated ORF108 protein. **, *P* < 0.01. (E) Binding of pET-28a-ORF108C-GFP and pET-28a-GFP proteins to WT, Δ*ompC*, and C-Δ*ompC* strains.

Different phages may utilize distinct bacterial surface structures as receptors. To investigate the presence of additional receptors on strain EDL933, we conducted lysis assays using deletion strains Δ*waaF* and Δ*etp* with other phages in our collection. Our results indicate that phage PNJ212 effectively lyses strain Δ*waaF* and Δ*etp*, suggesting that phage PNJ212 recognizes receptors on the surface of EDL933 beyond LPS and O antigen capsules. To identify additional phage receptors, we co-cultured phage PNJ212 with EDL933 in liquid LB medium. After the culture became turbid, we centrifuged the sample to collect the resistant bacterial population, from which genomic DNA was extracted for NGS. Mutation analysis revealed several genetic variations. We selected nine candidate receptor genes for deletion, but plaque assays indicated that only the *ompC* deletion strain exhibited resistance (Fig. 4; Fig. S5), suggesting that the *ompC* gene is involved in phage resistance. We subsequently generated a complementary strain C-Δ*ompC*, and plaque assays showed partial restoration of sensitivity (Fig. 4A). Phage lysis curve assays showed that the Δ*ompC* reduced sensitivity to phage PNJ212, while the C-Δ*ompC* regained sensitivity (Fig. 4B). Furthermore, the adsorption rate of phage PNJ212 to Δ*ompC* was significantly lower than that of the WT, confirming that OmpC serves as an additional receptor for phage PNJ212. We also identified the receptor types for phage PNJ212 adsorption. The results showed that the adsorption rate of the phage decreased after treatment with proteinase K, while it increased after treatment with sodium acetate (Fig. S6). This increase may be due to the removal of bacterial surface polysaccharides, which exposes more protein adsorption receptors. The results indicate that OmpC is the receptor for phage PNJ212 adsorption.

### Tail protein ORF108 in phage PNJ212 is an RBP that targets OmpC

We next conducted bioinformatic analyses to identify potential RBPs encoded by phage PNJ212. The protein ORF108 is predicted to function as a long tail distal protein and hypothesized to act as an RBP by interacting with the OmpC protein of *E. coli* strain EDL933. A C-terminal truncated variant of ORF108 (pET-28a-ORF108C) was successfully cloned and purified with a histidine tag. The purified tail fiber protein was incubated with the WT for 15 min, followed by the addition of phage PNJ212 and a further incubation for 10 min. After centrifugation, the phage titer in the supernatant was measured. The results revealed a reduction in the adsorption rate of phage PNJ212 to bacteria pre-incubated with the pET-28a-ORF108C compared to that of pre-incubated with BSA (Fig. 4D), indicating that the pET-28a-ORF108C competes for binding with bacterial surface receptors, thereby inhibiting phage adsorption. The interaction between the ORF108 protein and EDL933 was further examined using a fluorescence microscope. We expressed the GFP-tagged C-terminally truncated ORF108 protein (pET-28a-ORF108C-GFP) and incubated it with the WT, Δ*ompC*, and C-Δ*ompC* strains. As shown in Fig. 4E, ORF108C-GFP bound to both the WT and the complementary strain but not to the Δ*ompC* strain, confirming the interaction between ORF108 and OmpC.

### Adaptive costs of phage resistance

Next, we investigated how the evolution of phage resistance in bacteria affects their adaptability, focusing on different resistant strains.

**Fig 5:**
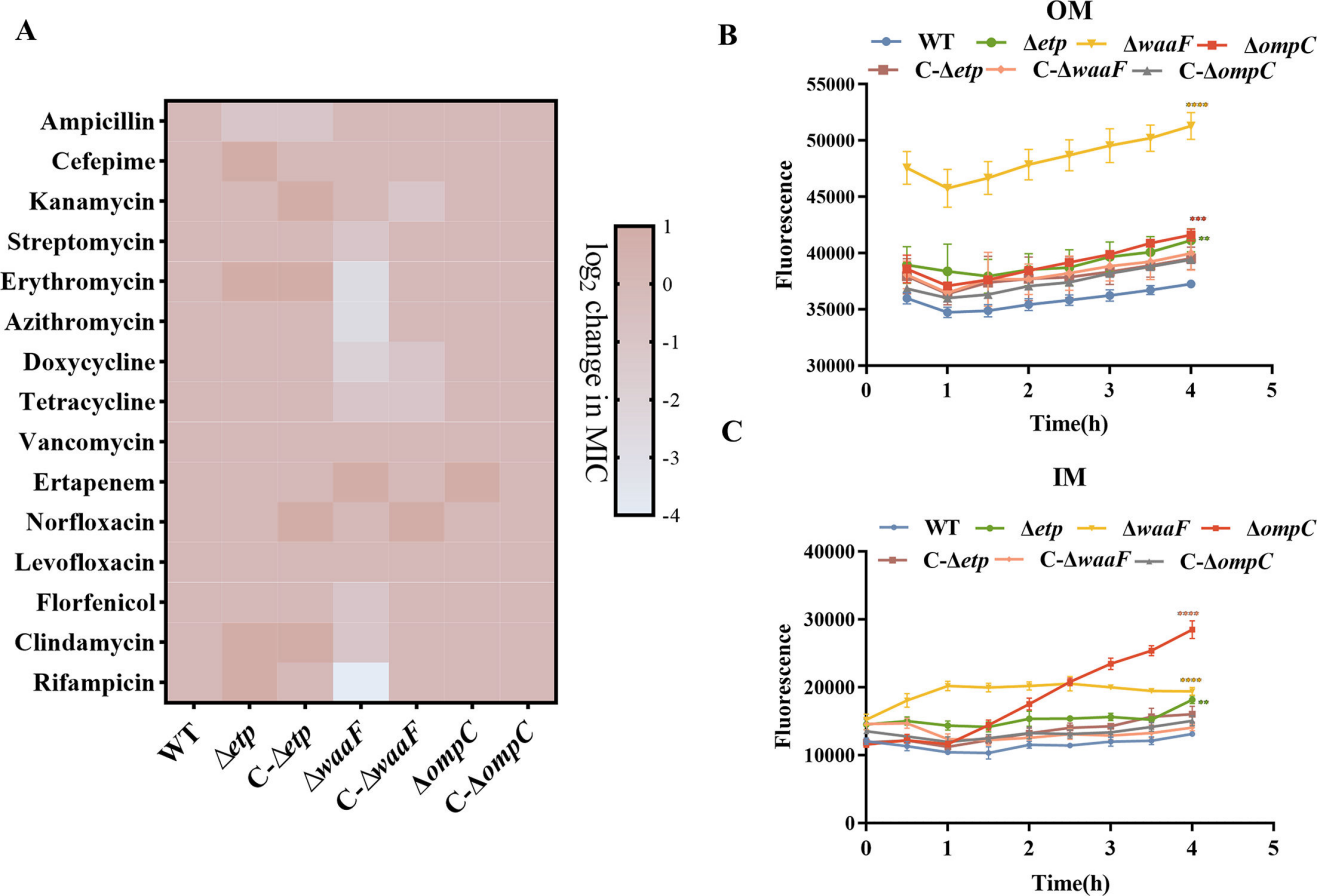
Antibiotic susceptibility assays and bacterial membrane permeability analysis. (A) Minimal inhibitory concentration (MIC) of 15 antibiotics measured using the microbroth-dilution method, with the log2 fold change in MIC compared between WT and phage-resistant strains. (B) Outer membrane permeability measurements for WT, Δ*waaF*, Δ*etp*, Δ*ompC*, C-Δ*waaF*, C-Δ*etp*, and C-Δ*ompC*. (C) Inner membrane permeability measurements for WT, Δ*waaF*, Δ*etp*, Δ*ompC*, C-Δ*waaF*, C-Δ*etp*, and C-Δ*ompC*. **, *P* < 0.01; ****, *P* < 0.0001.

We first assessed the antibiotic susceptibility of these resistant strains to 15 antibiotics, generating a heatmap to illustrate the changes in susceptibility (Fig. 5A). Notably, the Δ*waaF* deletion strain displayed a marked increase in susceptibility to erythromycin, azithromycin, and rifampicin, indicating that alterations in LPS structure may enhance antibiotic sensitivity. To further explore the underlying causes of increased sensitivity, we assessed the permeability of the inner and outer membranes. The results revealed a significant increase in permeability in the Δ*waaF* strain (Fig. 5B and C). Although the permeability of the inner and outer membranes was also elevated in the Δ*ompC* and Δ*etp* deletion strains (Fig. 5B and C), no significant changes in antibiotic sensitivity were observed.

**Fig 6:**
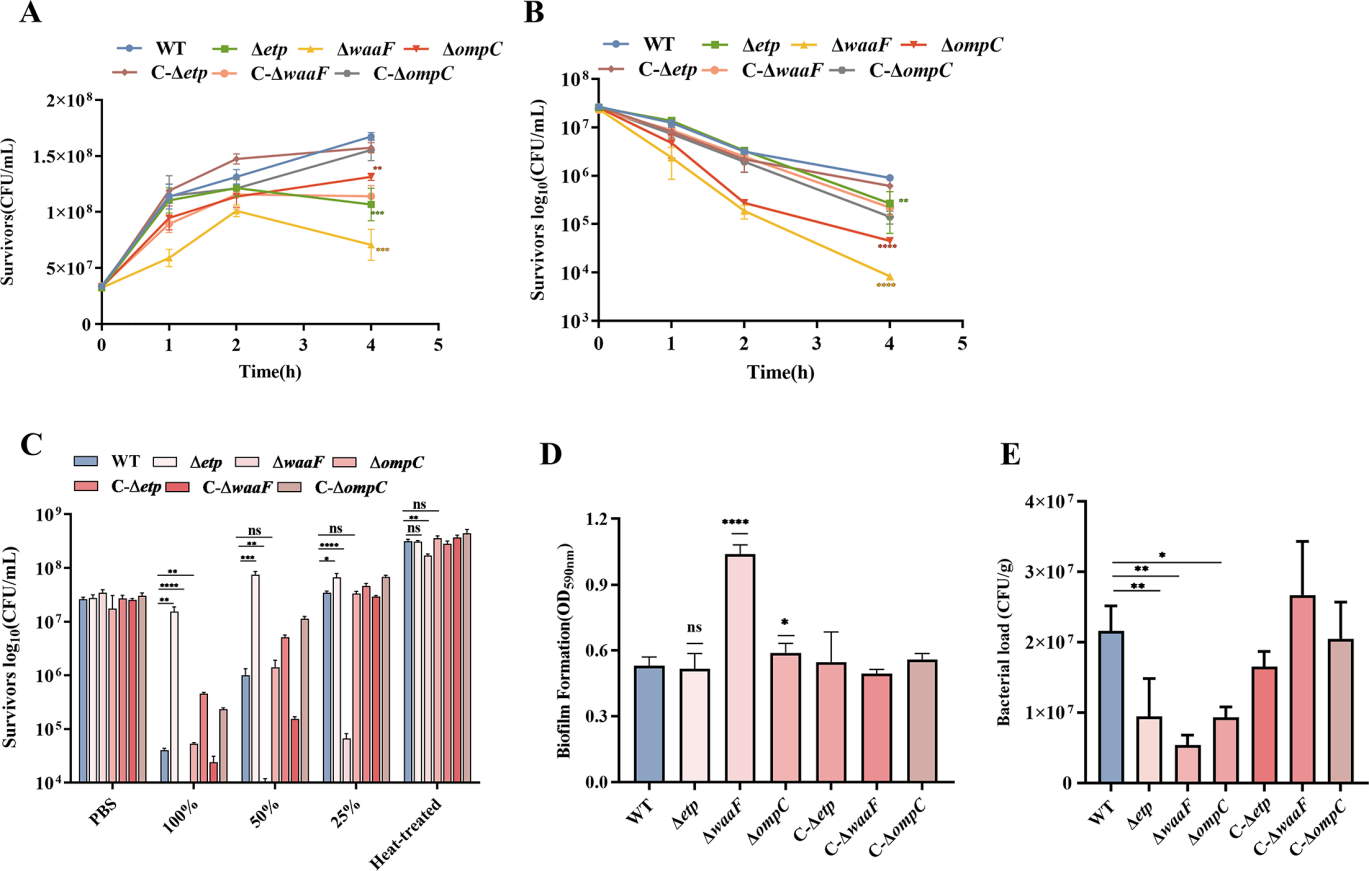
Fitness cost of phage-resistant bacteria.(A) Bacterial survival in simulated intestinal fluid for WT, Δ*waaF*, Δ*etp*, Δ*ompC*, C-Δ*waaF*, C-Δ*etp*, and C-Δ*o*mpC. (B) Bacterial survival in simulated gastric fluid for WT, Δ*waaF*, Δ*etp*, ΔompC, C-Δ*waaF*, C-Δ*etp*, and C-Δ*ompC*. (C) Serum survival assays for WT, Δ*waaF*, Δ*etp*, Δ*ompC*, C-Δ*waaF*, C-Δ*etp*, and C-Δ*ompC*. (D) Biofilm formation assays for WT, Δ*waaF*, Δ*etp*, Δ*ompC*, C-Δ*waaF*, C-Δ*etp*, and C-Δ*ompC*. (E) Bacterial load in the colon of SPF ICR mice for WT, Δwa*aF*, Δ*etp*, Δ*ompC*, C-Δ*waaF*, C-Δ*etp*, and C-Δ*ompC* in the colon of ICR mice. *, *P* < 0.05; **, *P* < 0.01; ***, *P* < 0.001; ****, *P* < 0.0001.

We next investigated the resistance of these mutant strains to simulated gastric and intestinal fluids and serum and their ability to form biofilms and colonize *in vivo*. In assays utilizing simulated gastric and intestinal fluids, the survival capacity of the Δ*waaF*, Δ*ompC*, and Δ*etp* strains was reduced to varying degrees, with the Δ*waaF* strain exhibiting the most significant reduction (Fig. 6A and B), indicating that phage-resistant bacteria exhibit reduced adaptability when exposed to these conditions. The results showed that the Δ*waaF* strain exhibited a significantly lower survival rate in human serum compared to the WT strain (Fig. 6C). In contrast, the Δ*etp* and Δ*ompC* strains demonstrated significantly higher survival rates than the WT strain (Fig. 6C). The loss of LPS compromises outer membrane integrity, making the bacteria more susceptible to the host immune response, which may explain the reduced survival in human serum. Furthermore, both the Δ*waaF* and Δ*ompC* strains exhibited a significant increase in biofilm formation, whereas Δ*etp* did not (Fig. 6D). Finally, in murine infection studies, the bacterial load in the colon was significantly lower in the mutant strains compared to the WT strain (Fig. 6E). Histopathological analysis of colon tissue sections stained with H&E revealed significant thinning of the colon wall and extensive damage, including fragmentation of intestinal villi, in the wild-type group compared to the control. In contrast, the mutant group exhibited relatively intact intestinal villi and milder pathological changes compared to the wild-type group. The complemented strain showed partial restoration, with intestinal villi damage and colon wall thinning resembling those observed in the wild-type group to varying degrees (Fig. 8). In summary, the results indicate that the LPS-deficient mutant represented by the Δ*waaF* strain incurs the highest fitness cost, while the CPS- and OmpC-deficient mutants exhibit varying degrees of fitness costs.

## DISCUSSION

The most common mechanism of bacterial resistance to phage is the alteration of surface adsorption receptors. Studying phage adsorption receptors is crucial for advancing the next generation of phage therapy, as understanding their function and localization can provide theoretical and strategic insights for optimizing phage-based treatments.

To identify the phage receptors of *E. coli* O157:H7 EDL933, we first focused on the highly lytic phage PSD2001 and conducted an in-depth analysis of its recognition mechanism. We identified the receptors of PSD2001 as the O antigen capsule and LPS, with key binding sites on the LPS located on Glc II, Glc III, and Gal I of the outer core polysaccharide. It has been reported that phages commonly use LPS as a receptor. The receptor for the *plague Yersinia*-specific phage P2 vir1 is primarily the terminal GlcNAc residue of the LPS outer core (26). The *Salmonella* phage P22 employs a tail spike protein to recognize the O antigen repeat units of LPS, which consist of one–six linked glucose units (27, 28). The LPS structure of EDL933 differs significantly from those of the aforementioned bacteria, and research on the key binding sites within its LPS remains limited. This study identifies the binding sites of PSD2001 as located on LPS Glc II, Glc III, and Gal I, which are adjacent to the outer core polysaccharide. This finding exemplifies the complexity of phage receptors, where T4-like phages utilize both O antigen capsules and LPS as dual receptors, with three distinct binding sites on LPS. Besides CPS and LPS, we identified OmpC as another receptor utilized by phage PNJ212 in the strain EDL933. Phage PNJ212 shares 96.59% genome sequence homology with PSD2001, both of which are T4-like phage. This finding underscores that even highly homologous phages may employ distinct mechanisms for host cell recognition, thereby enhancing our understanding of phage-host specificity.

T4 phage possess two sets of tail fibers and are known to be one of the most effective infection machines. The long tail fiber (LTF) is essential for the initial recognition of host cells and the initiation of infection. The LTF is composed of 10 polypeptide chains derived from four distinct gene products (gps): gp34 (140 kDa), gp35 (35 kDa), gp36 (23 kDa), and gp37 (109 kDa). The distal end of the LTF is formed by a homotrimer of gp36 and gp37, with the head structure of gp37 serving as the key element for host recognition and adsorption (29). We compared the C-terminal domain of ORF165 from PSD2001 and ORF108 from PNJ212 with the C-terminal domain of gp37 from various T-even phages, revealing significant homology (Fig. 7). Brzozozowska et al. demonstrated the interaction between T4 phage gp37 and the LPS of *E. coli* B using atomic force microscopy (30). The C-terminal protein of ORF165 from PSD2001 shares 16.22% homology with the C-terminal protein of gp37 from phage T4 and 66.66% homology with the C-terminal protein of gp37 from phage BP7. Correspondingly, the C-terminal protein of ORF108 from PNJ212 exhibits 62.45% homology with the C-terminal protein of gp37 from phage T4. Our results revealed that ORF165 from PSD2001 and ORF108 from PNJ212 are both capable of binding to their respective host receptors. Although both phage RBPs are gp37-like proteins, they share only 14.58% homology, and their adsorption receptors are completely distinct, potentially reflecting differences in their structural configurations.

Bacteria can acquire phage resistance by altering phage receptors, but this adaptation incurs a fitness cost. The Δ*waaF* strain exhibits increased sensitivity to certain antibiotics likely due to enhanced permeability of the bacterial inner and outer membranes. These antibiotics include erythromycin, azithromycin, clindamycin, tetracycline, and rifampicin, with erythromycin, azithromycin, tetracycline, and rifampicin classified as lipophilic drugs. We observed a significant increase in inner and outer membrane permeabilities in the Δ*waaF* strain likely due to severe defects in LPS structure, which compromise the barrier function of the cell membrane. This increase in permeability may contribute to the significantly enhanced sensitivity of the Δ*waaF* strain to certain antibiotics. The truncated LPS is referred to as deep rough mutants. Studies have shown that their permeability to lipophilic compounds is significantly higher than that of smooth strains. This increased permeability is attributed to the absence of the cationic cloud associated with the negatively charged LPS, which facilitates the passage of lipophilic drugs through the cell membrane and disrupts bacterial biosynthesis (31). The Δ*ompC* strain exhibited increased inner and outer membrane permeabilities, while its sensitivity to antibiotics remained unchanged. This lack of effect may result from the relatively minor disruption of the outer membrane caused by the absence of OmpC protein, with bacterial resistance mechanisms partially compensating for the changes in membrane permeability. OmpF-deficient mutants exhibit resistance to several antibiotics, including β-lactams, suggesting that OmpF is a major pathway for outer membrane permeability to these drugs. In contrast, OmpA is crucial for maintaining membrane integrity, and its absence in *ompA* mutants results in increased sensitivity to a broad range of antibiotics. Notably, OmpC plays a dual role (32). While deletion of the *ompC* gene can increase outer membrane permeability, compensation by the expression of other porins may mitigate the impact, such that the loss of OmpC does not significantly alter the overall antibiotic sensitivity of the bacteria. The impact of the Δ*etp* strain on outer membrane integrity appears minimal, with no significant effect on antibiotic sensitivity.

We assessed biofilm formation in phage-resistant bacteria and found that LPS-truncated strains exhibited increased biofilm formation. LPS truncation enhances the hydrophobicity of the bacterial cell surface, facilitating adhesion to polystyrene surfaces. Additionally, bacteria regulate biofilm formation through the production of extracellular DNA (33, 34). In this study, biofilm formation increased upon LPS truncation likely as a result of this mechanism. Additionally, bacteria tend to enter a self-aggregating state in response to significant structural defects, enhancing resistance to external threats and protecting their regulatory systems (35).

We investigated the survival capacity of mutant strains in both serum and simulated physiological fluids. Keiko’s previous studies demonstrated that in sepsis models, the survival rate of phage-resistant mutants of *E. coli* in serum was reduced (36). Similarly, the Δ*waaF* deletion strain exhibited decreased survival in serum. It has been reported that LPS mutants lacking O antigens are more susceptible to killing, as they have fewer virulence factors available to bind complement in serum (37). The absence of LPS compromises outer membrane integrity, making the bacteria more susceptible to the host immune response, which may contribute to reduced survival in human serum. The interaction between OmpC-specific antibodies and C1q is crucial for initiating antibody-dependent classical pathways, promoting the elimination of OmpC-expressing *E. coli*. Consequently, the absence of OmpC may allow these bacteria to evade immune clearance, resulting in prolonged survival in serum. The survival ability of Δ*etp* in serum was significantly enhanced compared to the WT likely due to the disruption of CPS synthesis, which promotes the synthesis of O antigens. O antigens provide serum resistance, thereby increasing the strain’s ability to withstand serum, consistent with the findings of Helen Miajlovic (38). The outer membrane serves as the primary barrier between bacteria and their environment, with CPS, LPS, and OmpC being key components. Their absence may impair the bacteria’s ability to resist gastric acid. It has been reported that the lack of O antigens in LPS reduces bile tolerance in *Salmonella* (39). OmpC and OmpF are critical for *E. coli* acid tolerance in the presence of arginine and lysine (40).

### Conclusion

This study elucidates the recognition mechanisms of two T4-like phages targeting the significant foodborne pathogen, *E. coli* O157:H7. Our findings reveal that even two phages with highly homologous genomes can recognize distinct receptors on the surface of their host bacteria. Notably, this study identifies that phage PSD2001 engages in multi-site binding to the LPS on the surface of *E. coli*, underscoring the complexity of phage-host interactions. It also demonstrates that while phage-resistant bacteria acquire resistance, the loss of LPS, CPS, and OmpC may impact bacterial adaptability through distinct mechanisms, resulting in varying adaptive costs. Attention should be focused on addressing phage resistance, particularly by exploiting bacterial adaptive deficiencies in conjunction with antibiotics or the immune system to effectively eliminate bacteria and overcome this challenge.

## MATERIALS AND METHODS

### Strains and growth conditions

The strains, plasmids, and phages used in this study are listed in Table S1. *E. coli* O157:H7 strain EDL933 originally isolated from raw hamburger meat and produced Shiga-like toxins I and II was used as the parental strain for developing phage-resistant mutants. Bacteria isolates were routinely grown at 37°C on solid or in solid Luria-Bertani (LB).

### Phage preparation

PSD2001 and PNJ212 were isolated from duck manure in 2020 and propagated in EDL933. A mixture of EDL933 and either PSD2001 or PNJ212 in 3 mL of 0.5% LB was plated onto LB agar and incubated at 37°C for 10 h. After incubation, plates exhibiting confluent lysis were flooded with 5 mL of sterile SM buffer [0.05 M Tris-Cl (pH7.5), 5.8 g NaCl, 2.0 g MgSO₄·7H₂O, and 5 mL of 2% (wt/vol) gelatin solution]. The plates were then incubated at 4°C overnight. Phage and cell suspensions were harvested and filtered through a sterile 0.22 µm membrane filter. Phage stocks were stored in SM buffer at 4°C.

### Selection of phage-resistant mutants

EDL933 was inoculated into 5 mL of LB liquid culture medium at a 1:100 ratio and cultured in logarithmic phase at 180 rpm and 37°C. PSD2001 (10⁸ PFU/mL) was mixed with the EDL933 at a 1:1 volumetric ratio and incubated at 37°C for 10 min. Then, 5 mL of preheated semi-solid LB medium was added, mixed, poured onto solid LB plates, and incubated overnight at 37°C. Colonies resistant to the incubator conditions were selected using the double-agar overlay method. PNJ212 and EDL933 were mixed in a 1:1 ratio and inoculated into 10 mL of liquid LB medium. The culture was incubated for 6 h at 37°C, followed by centrifugation to collect the bacterial cells.

### DNA sequencing and bioinformatics analysis of mutation sites

Genomic DNA was extracted and purified using a bacterial DNA extraction kit (Omega, USA) and sequenced on an Illumina NovaSeq PE150 platform. Libraries were prepared using the Illumina TruSeq Nano DNA Sample Prep Kit. Raw sequencing data were filtered using Trimmomatic software to obtain high-quality sequencing reads (clean data). The resulting reads were aligned to the reference genome using BWA software. PCR duplicates were removed using Picard tools. SNP and small indel detection was performed using the GATK toolkit, followed by filtering to obtain high-confidence variants based on the following criteria: (i) removal of non-biallelic sites, (ii) SNP quality ≥ 20, (iii) SNP depth ≥ 4, and (iv) QD ≥ 2.0, FS ≤ 60.0, MQ ≥ 40.0, MQRankSum ≥ −12.5, and ReadPosRankSum ≥ −8. SNP detection results were annotated using an in-house Perl script.

### Construction of gene deletion and complementation mutants

Gene deletion strains were constructed using lambda red homologous recombination primers (41). First, 50 bp homologous arms were designed for both the upstream and downstream regions of the target gene. Kanamycin-resistant plasmids were used as amplification templates. The pKD46 plasmid conferring kanamycin resistance was transformed into the deletion plasmid construct. Primers (K1, K2, and downstream) were used to verify the successful recombination, as detailed in Table S2. Next, the pCP20 plasmid was introduced via electroporation to remove the kanamycin resistance cassette and generate traceless strains. For the construction of deletion strains, target primers were designed to amplify the desired fragments, and the pSTV28 plasmid was used for substitution, facilitating the introduction of the fragments into the strains. The primers used in this process are listed in Table S2.

### Phage spot assay

A 100 µL sample of bacterial culture in logarithmic phase was mixed with 5 mL of semi-solid LB medium and evenly spread onto the surface of solid LB agar. Once the semi-solid LB medium had solidified, 10 µL of serially diluted phage culture (diluted serially to 10⁻¹, 10⁻², 10⁻³, 10⁻⁴, and 10⁻⁵) was applied dropwise to the previously marked areas on the plate. After allowing the drops to dry, the plate was incubated at 37°C in a constant temperature incubator.

### Lysis curve measurement

The lysis curve was measured by a microplate reader (TECAN, Switzerland). A 100 µL sample of bacterial culture in logarithmic phase was centrifuged for 8 min at 8,000 ×*g*. The bacterial pellet was washed three times with LB medium and resuspended. Next, 100 µL of the bacterial culture was mixed with 100 µL of the phage or LB liquid medium in a 96-well plate. The 96-well plate was then incubated at 37°C with shaking at 180 rpm, with OD_600_ values recorded every 30 min for 8 h of continuous monitoring.

### Adsorption assays

A 10 µL sample of phage (10⁷ PFU/mL) was added to 500 µL of bacterial culture in logarithmic phase. The negative control group consisted of 500 µL of LB liquid medium mixed with 10 µL of phage. The mixture was incubated at 37°C for 10 min, followed by centrifugation at 4°C for 10 min. After centrifugation, the supernatant was collected, and phage titer was determined using the double-layer agar plate assay. Phage adsorption efficiency was calculated using the formula: Phage adsorption efficiency (%) = [1 − (phage titer in the supernatant after adsorption / phage titer in the control without bacteria)] ×100.

### Adsorption inhibition assays

A 500 µL sample of bacterial culture was combined with 500 µL of phage suspension (10⁴ PFU/mL) and 10 µL of LPS solution at varying concentrations (Alpha Diagnostic International). The mixture was incubated at 37°C for 10 min, and then centrifuged at 10,000 ×*g* for 10 min at 4°C. The phage adsorption rate was subsequently measured.

### Capsule observation

The capsule of WT, Δ*etp*, and C-Δ*etp* strains was analyzed using three methods: colony morphology, capsule staining, and electron microscopy. Bacterial colonies grown on solid LB medium for 16 h were observed under an inverted microscope (Leica DMI 1) to assess overall morphology. For capsule staining, a small number of colonies were suspended in ddH₂O, smeared onto a slide, air-dried, and fixed with 95% alcohol. The fixed smear was stained with violet dye for 1 min, rinsed with water, and air-dried. Ink was then spread along the smear’s edge to create a film, which was visualized under an upright microscope (Leica DM500). For high-resolution observation, electron microscopy was performed by transferring a bacterial suspension onto a copper mesh pre-coated with phosphate-buffered saline (PBS). The sample was negatively stained with 2% phosphotungstic acid (wt/vol) and imaged using a transmission electron microscope (Hitachi H7650).

### Identification of receptor types

The bacterial culture in the logarithmic phase was centrifuged at 8,000 ×*g*, and the pellet was washed three times with PBS. The pellet was then incubated for 1 h with liquid LB medium, proteinase K, or sodium periodate. After incubation, the bacteria were washed by centrifugation and resuspended in PBS. The adsorption assay for treated bacteria was conducted using the aforementioned method.

### Expression of C-terminal protein ORF165 and verification of its binding to LPS

To assess whether the C-terminal truncated tail protein ORF165 of phage PSD2001 can bind to the LPS of EDL933, the vector pET-28a-ORF165C was constructed, and the protein was purified using a HisTrap FF column (GE Healthcare BioSciences AB, Sweden). Purified tail fiber protein at different concentrations was incubated with commercial LPS *in vitro* for 1 h, with HOC protein as a control. Equal volumes of phage were then added to the incubation mixture and incubated for an additional hour. Phage titer was determined using the double-layer agar plate assay after incubation.

### Competitive binding assay for C-terminal of ORF108

The vector pET-28a-ORF108C was constructed, and the protein was purified using a HisTrap FF column. A 100 µL sample of EDL933 bacterial suspension was mixed with 100 µL of purified tail fiber protein (0.5 mg/mL) or LB medium and incubated at 37°C for 15 min. Phage propagation solution was then added, and the mixture was incubated at 37°C for an additional 10 min. After centrifugation at 10,000 ×*g* for 5 min, the phage titer in the supernatant was determined for both groups.

### Binding assays for the C-terminal of ORF108 to OmpC

To investigate the interaction between the tail fiber protein ORF108 of phage PNJ212 and the OmpC receptor in EDL933, a recombinant construct encoding the C-terminal domain of ORF108 fused to GFP was generated (pET-28a-ORF108C-GFP). A control plasmid expressing GFP (pET-28a-GFP) was also constructed. The expressed fluorescent fusion proteins were purified using a HisTrap FF column. For binding assays, 100 µL of purified pET-28a-ORF108C-GFP or pET-28a-GFP protein (1 mg/mL) was incubated with 100 µL of bacterial suspensions (10⁸ CFU/mL) of WT, Δ*ompC*, or C-Δ*ompC* strains at 37°C for 20 min. Following incubation, unbound proteins were removed by washing the bacterial cells three times with PBS. The cells were then mounted on slides and examined using a ZEISS Axio Observer inverted fluorescence microscope equipped with ZEN software at 100× oil immersion to assess protein binding.

### Minimum inhibitory concentration (MIC)

Antibiotic stock solutions (2.56 mg/mL) were diluted in cation-adjusted Mueller-Hinton broth to the desired working concentrations. Bacterial cultures were grown at 37°C with shaking to the logarithmic phase and adjusted to ~2 × 10⁵ CFU/mL. A total of 100 µL of each antibiotic concentration and 100 µL of diluted bacterial suspension were added to the wells of a 96-well plate and incubated at 37°C for 18 h.

### Inner and outer membrane permeability assays

For the inner membrane permeability assay, log-phase bacteria were washed three times with PBS and adjusted to an OD of 0.5. A 190 µL sample of bacterial suspension was mixed with 10 µL of propidium iodide (PI) solution (20 µg/mL) and transferred to a 96-well plate. Fluorescence was continuously monitored using a microplate reader with excitation and emission wavelengths of 535 and 615 nm, respectively. For the outer membrane permeability assay, log-phase bacteria were similarly washed and adjusted to an OD of 0.5. A 198 µL sample of bacterial suspension was mixed with 2 µL of NPN (1 mM) and added to a 96-well plate. Fluorescence was continuously measured with excitation and emission wavelengths of 350 and 420 nm, respectively.

### Serum-mediated bactericidal activity assay

Serum was diluted to final concentrations of 100, 50, and 25%. Serum was inactivated by heating in a 56°C water bath for 30 min. A 40 µL of bacterial suspension (10⁷ CFU/mL) was mixed with 360 µL of serum at specified concentrations (100, 50, and 25%) or with heat-inactivated serum and incubated at 37°C for 2 h. The number of survival bacteria was quantified by plate counting. Bacteria incubated in PBS served as the control.

### Stability of different strains in simulated gastric fluid (SGF) and simulated intestinal fluid (SIF)

SGF was prepared with 0.13 g NaCl, 0.64 g NaHCO₃, 0.024 g KCl, and 1 g pepsin in 100 mL of distilled water (pH 2.5), and SIF was prepared with 0.68 g KH₂PO₄ and 1 g sterile pepsin in 100 mL of water (pH 6.8). The bacterial culture was diluted to 10⁷ CFU/mL and mixed with 900 µL of SGF or SIF. The mixture was incubated at 37°C with shaking at 180 rpm. At each time point (0, 1, 2, and 4 h), a 100 µL sample was collected and serially diluted 10-fold, and the number of survival bacteria was quantified by plate counting.

### Determination of colonic bacterial load

To determine the bacterial colonization ability in the colonic, the bacterial strain was inoculated into LB broth and incubated at 37°C with shaking at 180 rpm for 3 h. The bacteria were then collected by centrifugation, washed three times with PBS, and resuspended in PBS to a concentration of 10⁹ CFU/mL. SPF ICR mice were orally gavaged with 100 µL of the bacterial suspension, while the control group received 100 µL of PBS. After 24 h, the mice were euthanized, and the colons were collected and homogenized. The homogenates were plated on sorbitol MacConkey agar. Additionally, colon sections were prepared and stained with hematoxylin and eosin (H&E) for histological analysis.

### Data analysis

The data are presented as means ± standard deviations from a minimum of three independent experiments. Statistical analyses were performed using Student’s *t*-tests with GraphPad Prism v7 software. A *P*-value ≤ 0.05 was considered statistically significant. Significance levels are indicated in the figures by asterisks (*, *P* < 0.05; **, *P* < 0.01; ***, *P* < 0.001; ****, *P* < 0.0001).

## Data Availability

Annotated phage and bacteria were deposited in NCBI/ENA/DDBJ and assigned the following accession numbers: OK254198.1 (PSD2001), PQ765507 (PNJ212), SRR25083116 (*E.coli* O157: H7 EDL933), SRR25083115 (SD0102), SRR25083114 (SD0123), SRR25083113 (SD0126), and SRR31366891 (bacteria resistant to PNJ212).

## SUPPLEMENTAL MATERIAL

Supplemental figures spectrum.00067-25-s0001.docx Fig. S1 to S8.

Table S1 spectrum.00067-25-s0002.docx Bacterial strains and plasmids.

Table S2 spectrum.00067-25-s0003.docx Primers used.

ASM does not own the copyrights to Supplemental Material that may be linked to, or accessed through, an article. The authors have granted ASM a non-exclusive, world-wide license to publish the Supplemental Material files. Please contact the corresponding author directly for reuse.
