# Supplementary material for: Multisite binding of bacteriophages on lipopolysaccharides in Escherichia coli O157:H7 and the adaptive costs of phage resistance
Source: Microbiol Spectr. 2025 Jun 17;13(8):e00067-25. doi: 10.1128/spectrum.00067-25 (PMC12323348; doi:10.1128/spectrum.00067-25)
Supplement: Supplemental figures — Fig. S1 to S8. [file spectrum.00067-25-s0001.docx]

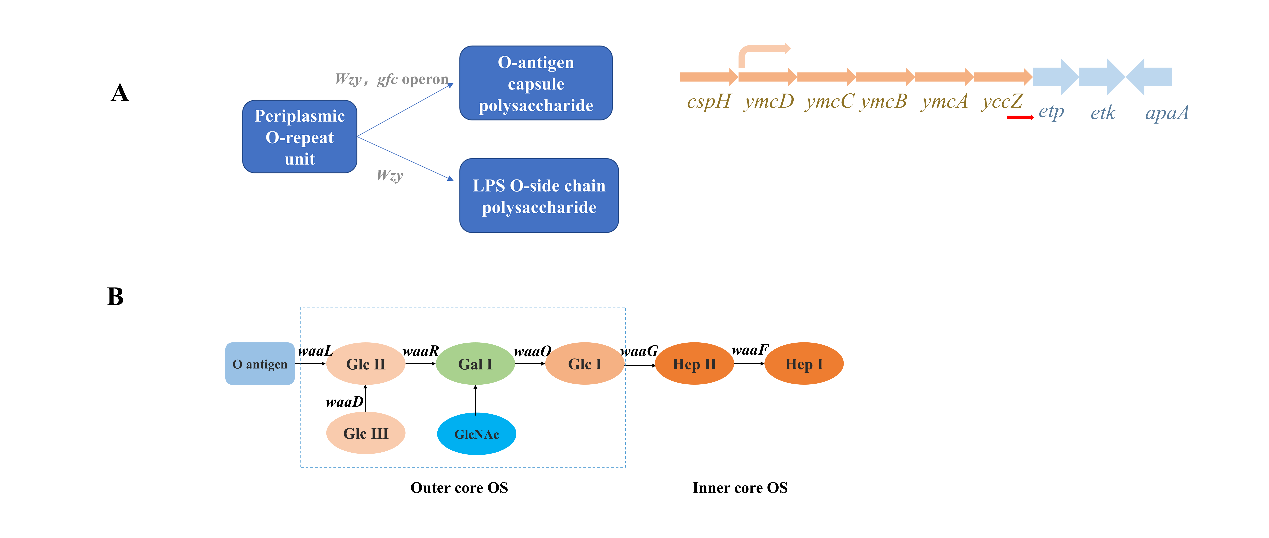


**FIG S1** **Gene cluster diagram of the LPS and capsule.** (A) Conserved capsular gene cluster in EDL933. (B) Schematic representation of the core polysaccharides synthesis process in LPS. Distinct colors denote different oligosaccharides, while the dashed box highlights the composition and synthesis direction of the outer core polysaccharide.


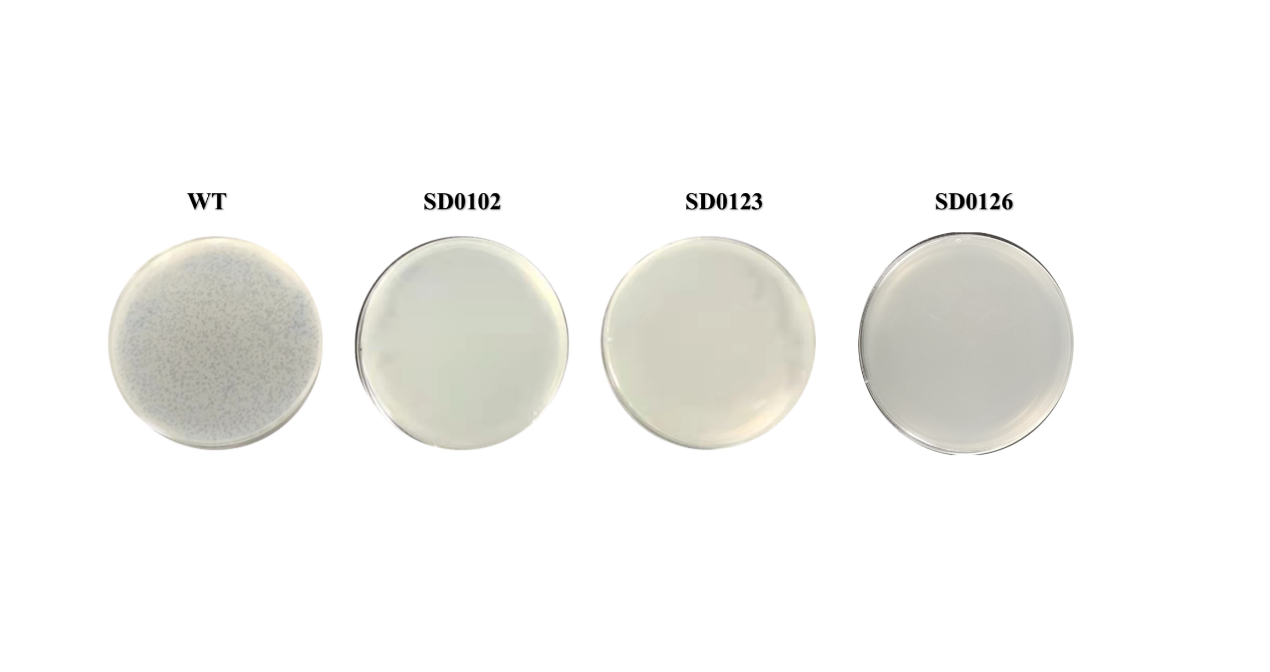


**FIG S2** Susceptibility of WT and mutant strains to phage PSD2001 assessed using the double-layer agar method.


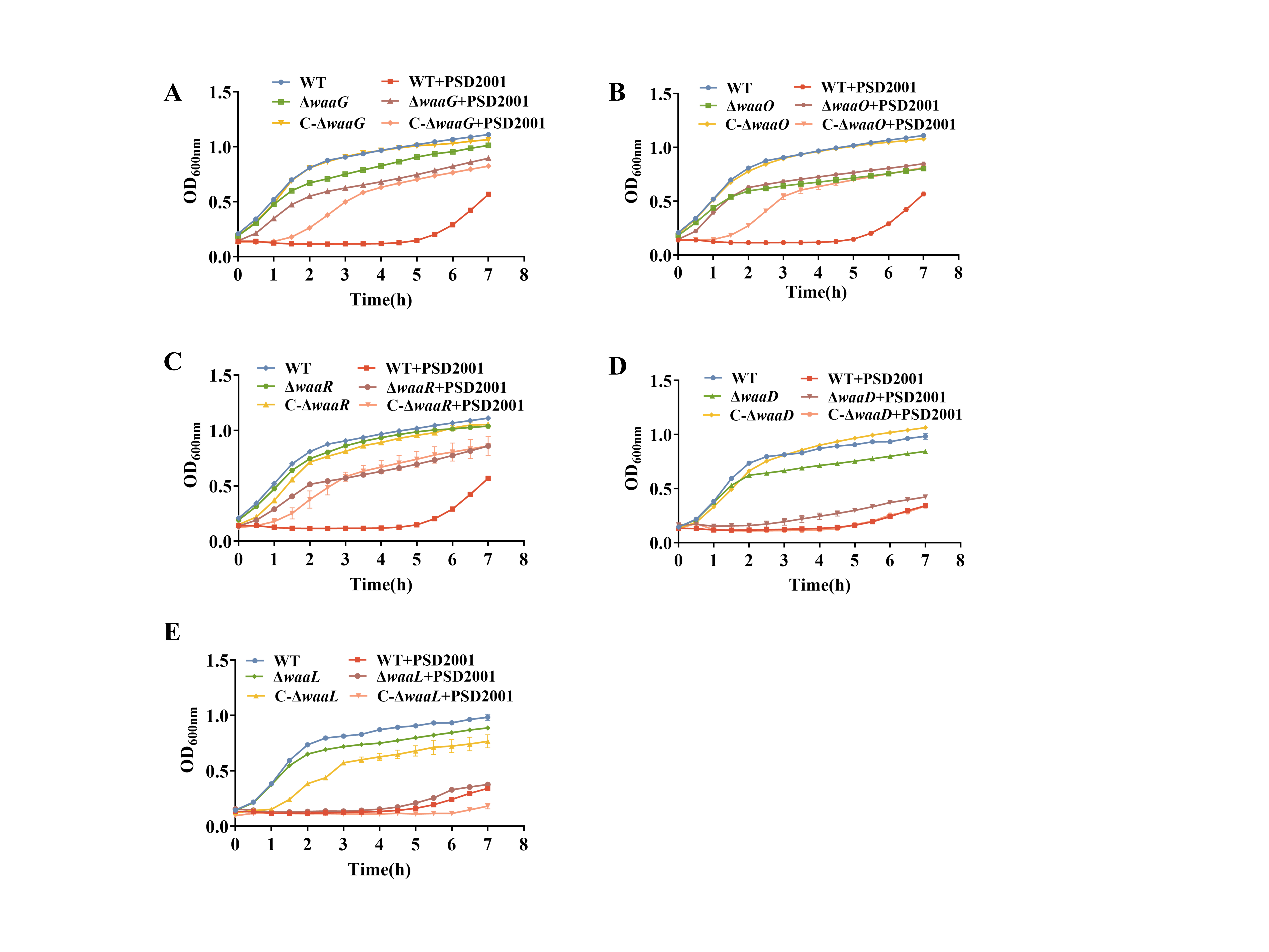


**FIG S3 Lysis curve of WT, deletion and complementary strains.**


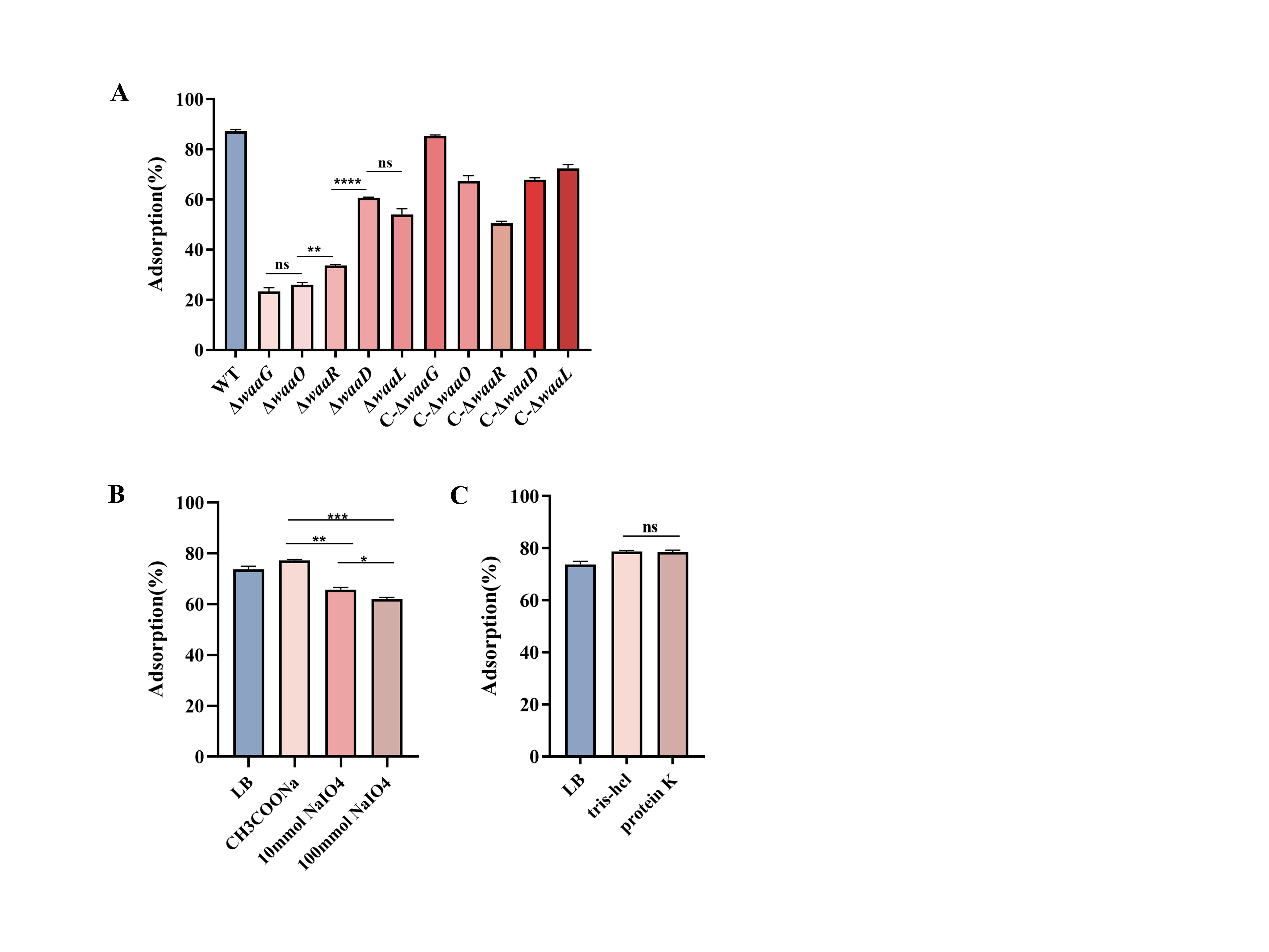


**FIG S4** **Adsorption rates analysis of phage PSD2001.**

(A) Adsorption rates of phage PSD2001 in WT and various gene deletion mutants; (B) Adsorption rates of phage PSD2001 in strains treated with LB medium (negative control); 50 mM CH3COONa (pH 5.2); 10 mM sodium periodate (NaIO4) and 50 mM sodium acetate (CH3COONa) (pH 5.2); 100 mM NaIO4 and 50 mM CH3COONa (pH 5.2).(C) Adsorption rates of phage PSD2001 in strains treated with LB medium (negative control); 20 mM tris-hcl and 100 mM NaCl (pH 7.5); 0.5 mg/ml proteinase K in 20 mM tris-hcl and 100 mM NaCl (pH 7.5). After incubation of the stains with phage PSD2001, free virions in the supernatant were quantified using plaque assays to determine the adsorption rates. LB medium served as a negative control. Protein K was used to degrade protein receptors, while sodium periodate was employed to disrupt polysaccharide receptors. *, *P* < 0.05; **, *P* < 0.01; ***, *P* < 0.001; ****, *P* < 0.0001.


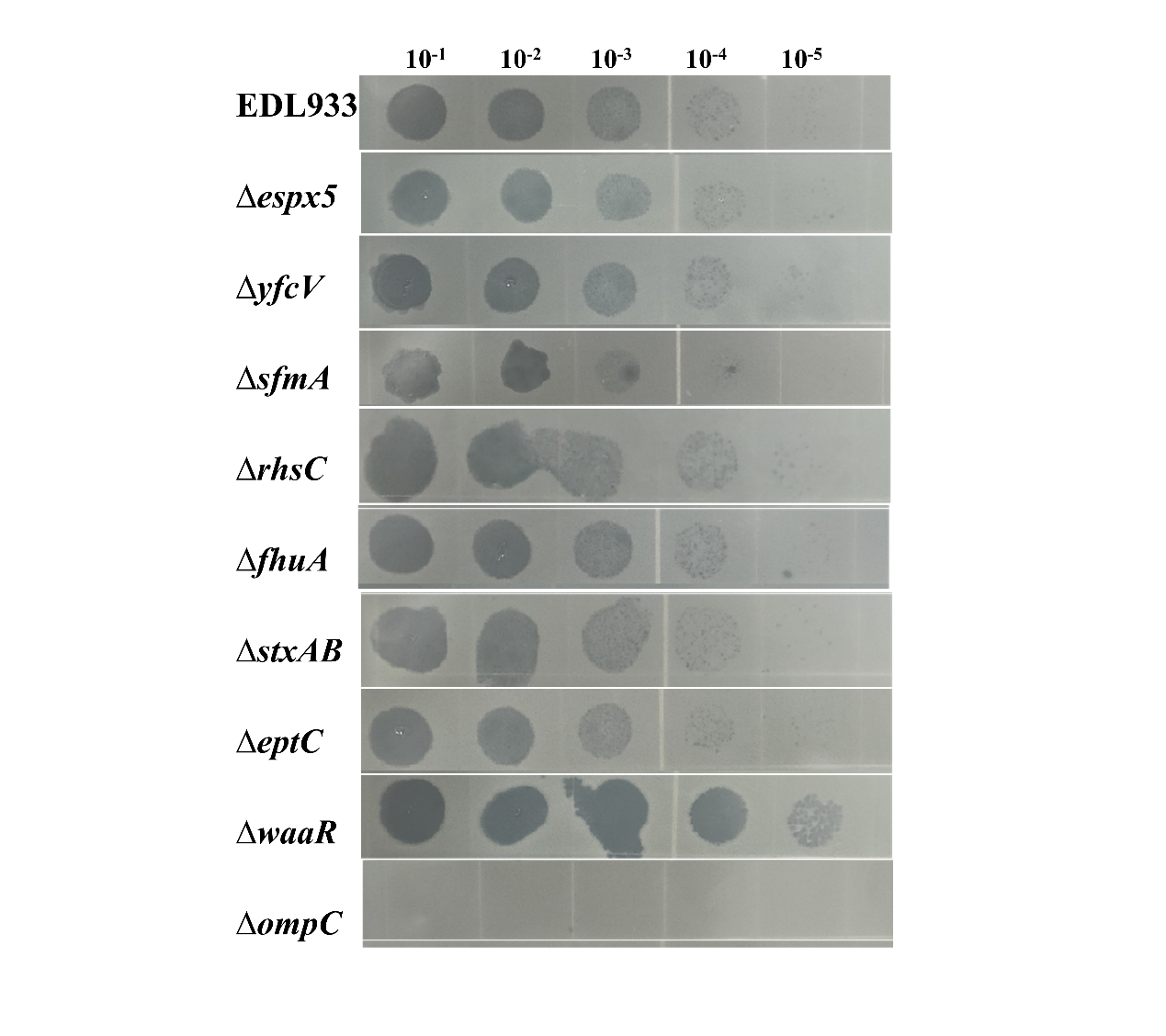


**FIG S5 Spot assays.** The spot assays were conducted by applying continuously diluted phage PNJ212 solutions onto plates inoculated with different bacterial strains.


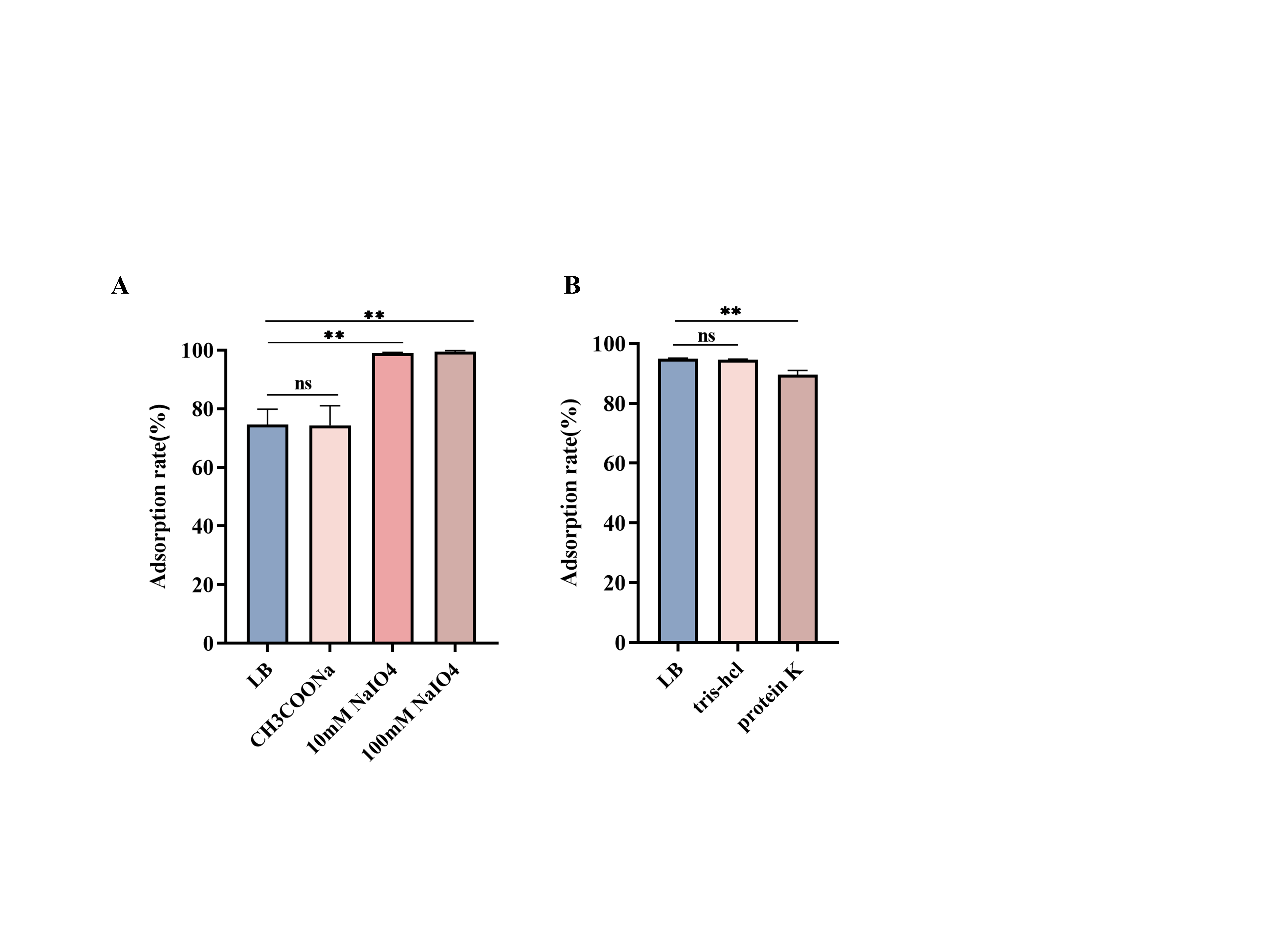


**FIG S6 Adsorption rates of phage PNJ212 to host EDL933 treated with various agents**. Adsorption rates of phage PNJ212 to host E. coli EDL933 were evaluated following treatment with different agents. LB medium was used as a negative control. Proteinase K was applied to degrade protein receptors, and sodium periodate was used to disrupt polysaccharide receptors. **, *P* < 0.01.


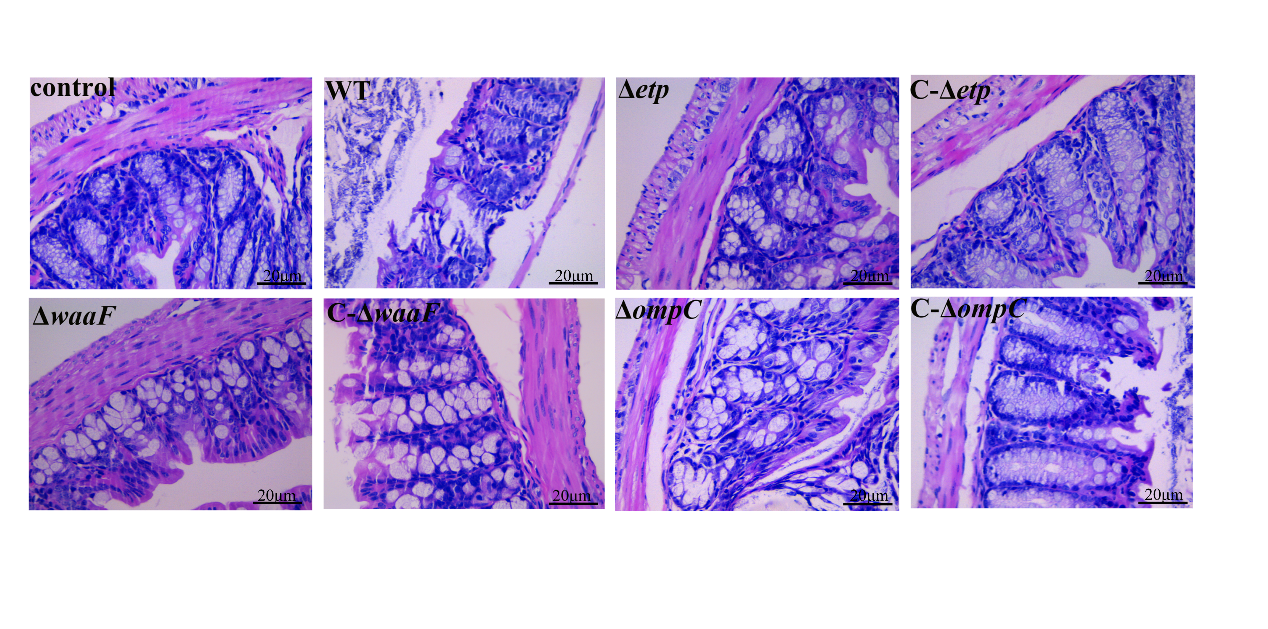


**FIG S7** **H&E staining of histological sections of the colon.**

Colon samples were collected 24 h after intragastric administration of PBS or bacteria in mice. Scale bars:20µm.


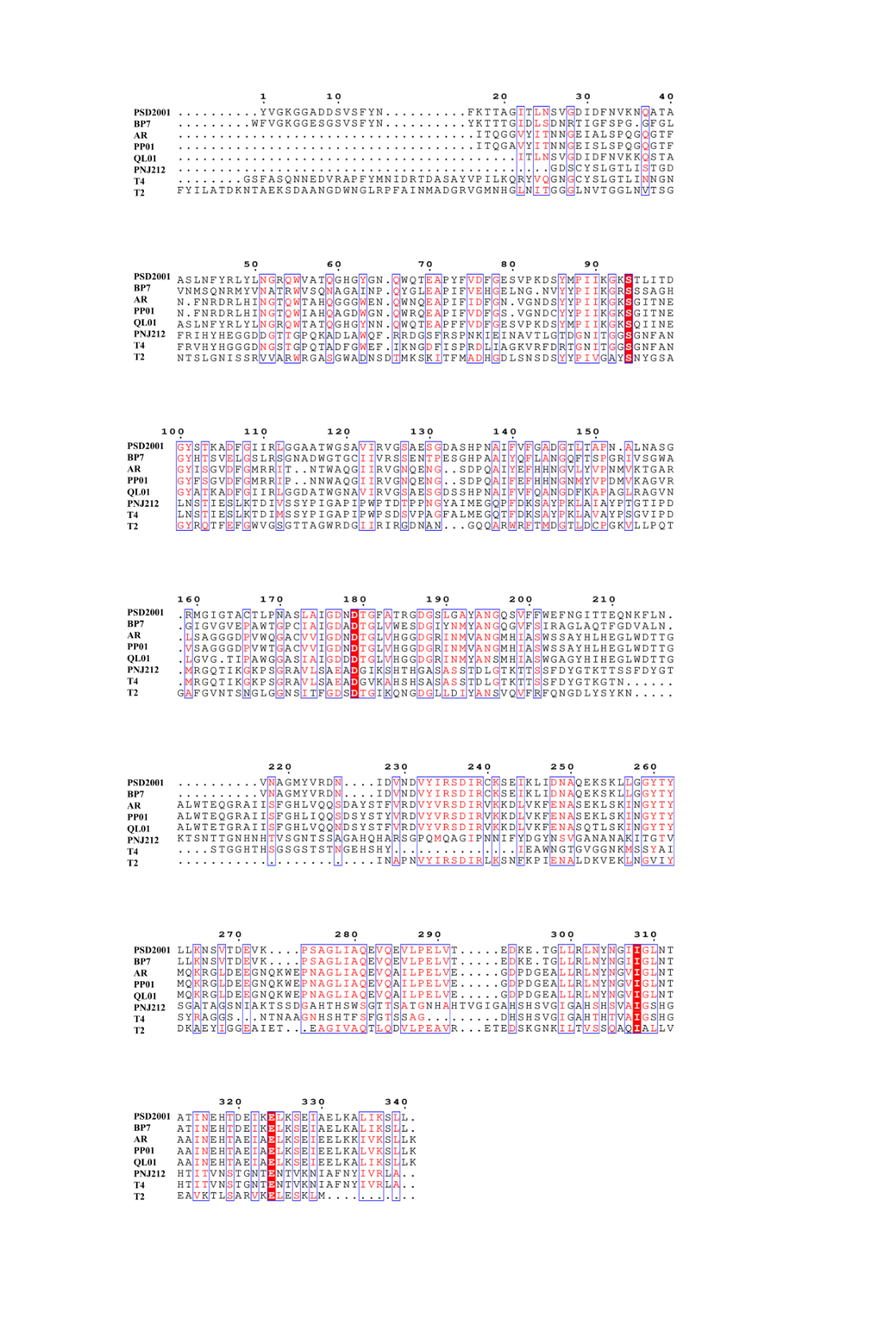


**FIG S8 Alignment of C-terminal sequence among protein ORF165 of PSD2001, ORF108 of PNJ212 and gp37 of six additional phages.**

Blue boxes indicate highly conserved residues,while residues conserved across all aligned sequences are highlighted with a red background.
