# Supplementary material for: Multisite binding of bacteriophages on lipopolysaccharides in Escherichia coli O157:H7 and the adaptive costs of phage resistance
Source: Microbiol Spectr. 2025 Jun 17;13(8):e00067-25. doi: 10.1128/spectrum.00067-25 (PMC12323348; doi:10.1128/spectrum.00067-25)
Supplement: Table S1 — Bacterial strains and plasmids. [file spectrum.00067-25-s0002.docx]

| Strains or plasmid | Relevant characteristics or description |
| --- | --- |
| Strains |  |
| DH5a | F^-^Δ(*lacZYA*-*argF*)*U169* *recA1 endA1 hsdR17*(r_K_^-^ 2 m_K_^+^ )*phoA supE44* λ^-^ |
| BL21(DE3) | F^-^ *ompT* *hsdS*(r_B_^-^ mB^-^) *gal dcm* (DE3) |
| EDL933 | *E.coli* O157：H7 Wild-type strain，host bacterium of phage PSD2001 and PNJ212 |
| Δ*etp* | Deletion of *etp* from EDL93358 |
| C-Δ*etp* | Δ*etp*-pSTV28-*etp* |
| Δ*waaF* | Deletion of *waaF* from EDL933 |
| C-Δ*waaF* | Δ*waaF*-pSTV28-*waaF* |
| Δ*hrpB* | Deletion of *hrpB* from EDL933 |
| Δ*ompC* | Deletion of *ompC* from EDL933 |
| C-Δ*ompC* | Δ*ompC*-pSTV28-*ompC* |
| Phage |  |
| PSD2001 | *E. coli* phage |
| PNJ212 | *E. coli* phage |
| Plasmids |  |
| pKD46 | Amp，expresses λ red recombinase |
| pKD4 | *kan* gene，template plasmid |
| pCP20 | Cm，Amp，yeast Flp recombinase gene，FLP |
| psTV28 | Cm，*lacZ* |
| pET28a | Kan，F1 origin，His tag |
| pEGFP-N2 | Kan，amplify the EGFP gene for constructing a protein expression vector |

**TABLE S1** Bacterial strains and plasmids
