# Supplementary material for: Multisite binding of bacteriophages on lipopolysaccharides in Escherichia coli O157:H7 and the adaptive costs of phage resistance
Source: Microbiol Spectr. 2025 Jun 17;13(8):e00067-25. doi: 10.1128/spectrum.00067-25 (PMC12323348; doi:10.1128/spectrum.00067-25)
Supplement: Table S2 — Primers used. [file spectrum.00067-25-s0003.docx]

**TABLE S2** Primers used in this study

| Primer | Sequence (5’–3’) |
| --- | --- |
| Amplification of the target gene for deletion | |
| F-Δ*waaF* | GTGTAACGGAATACATGGCCTGGCTGAATCGCGACGCATAAGAGCTCTGCGTGTAGGCTGGAGCTGCTTCGA |
| R-Δ*waaF* | TGGAGAACATCGCCCATCGACGATGTTTTAACGATCAAAACCCGCATCCGCATATGAATATCCTCCTTAG |
| F-Δ*hrpB* | CCGCGATGCTACAATGTGGCGCGAAGAATGTTAACCCTCTGGAGCGTTTTGTGTAGGCTGGAGCTGCTTCGA |
| R-Δ*hrpB* | TGGTGCCCTCACCCCGGCCCTCTCCCACAGGGAGAGGGAGAAAACCAACCCATATGAATATCCTCCTTAG |
| F-Δ*etp* | CTGGTGGCGTATTCATATTTTTAGTTGTCATCGTATTTTATAAACTCAAAGTGTAGGCTGGAGCTGCTTCGA |
| R-Δ*etp* | ACGATTTCCGGGGTGCATGACCTGACAGAGACTTCACGGTGGATTCAAACCATATGAATATCCTCCTTAG |
| F-Δ*waaG* | CCTCAAAAGGATCTTTACCGCGCCATAACGTGGCAAACGGCTCTTTAAGTGTGTAGGCTGGAGCTGCTTCGA |
| R-Δ*waaG* | ATTGCTGCTGTCGATAAATTACTGCCCTCCTCCACGACAGGTACGTCGTTCATATGAATATCCTCCTTAG |
| F-Δ*waaO* | CATTTATCGTTTTATTATATATCATAATGAATTATTTTAACCTTAAATCTGTGTAGGCTGGAGCTGCTTCGA |
| R-Δ*waaO* | CGTTGTAACCCGTAACTTATTTTTGCCAAAATTTTGGATACAGAATAAATCATATGAATATCCTCCTTAG |
| F-Δ*waaR* | TGAAAAAATAGGAGTAACCGTAAATATTATTTTATCAACCATCTCGTATAGTGTAGGCTGGAGCTGCTTCGA |
| R-Δ*waaR* | GATGTAAAAGTCAAACTAGGATTGAAGAGCAAATAATGATTATAATGAAGCATATGAATATCCTCCTTAG |
| F-Δ*waaD* | ATGAAAAACAAGTAAAAAATAAAAAAGGCTGCATAATGCGGCCTTTTTATGTGTAGGCTGGAGCTGCTTCGA |
| R-Δ*waaD* | GGTGTTTTTACAGCAATTAAATATAATGTTATGAAAGGTTAATATACGAGCATATGAATATCCTCCTTAG |
| F-Δ*waaL* | CAAAATTTAAATCTTGAAATAATAACCAATAAGTTGACATCGGAGATAAGGTGTAGGCTGGAGCTGCTTCGA |
| R-Δ*waaL* | AATTGGTTTGAATAAATAAAAAGGCCGCATTATGCAGCCTTTTTTATTTTCATATGAATATCCTCCTTAG |
| F-Δ*ompC* | TGCAGTGGCATAAAAAAGCAAATAAAGGCATATAACAGAGGGTTAATAACTGTAGGCTGGAGCTGCTTCG |
| R-Δ*ompC* | AAAACAATGAAAAAAGGGCCCGCAGGCCCTTTGTTCGATATCAATCGAGAATATGAATATCCTCCTTAG |
| Recombinant vector identification | |
| K1 | CAGTCATAGCCGAATAGCCT |
| K2 | CGGTGCCCTGAATGAACTGC |
| *waaF* up | GGATAAGCTGAAAGGCCGCTA |
| *waaF* down | TTGCCACAGGAATAACTCGCT |
| *hrpB* up | GCAAAGAACAGACGTTGCGG |
| *hrpB* down | ATATTCAGGCGCAAAAGCCC |
| *etp* up | ACGCGGTCACGCTGATAATA |
| *etp* down | AGGACGCATCGGCAATGATA |
| *waaG* up | CTTTACCGCGCCATAACGTG |
| *waaG* down | TGTTTGGTGCGACAGACCAT |
| *waaO* up | GGTCATTTTATGCTTTGGCG |
| *waaO* down | CAAAAATCAGGGAAAGAACG |
| *waaR* up | GAGGAGCTCGCTTACGAATT |
| *waaR* down | AGATCGCATTGATCTTGAAC |
| *waaD* up | CTGGTTTGGCACTGGTTTAT |
| *waaD* down | CATGATTGGGCGAACTACGC |
| *waaL* up | GCGGAAGGATTTGCTTATGT |
| *waaL* down | GGTCAGGACTTTGTGTTTGA |
| *ompC* up | CCGACTGATTAATGAGGGTT |
| *ompC* down | TGATTATCCTCATGCGAACG |
| Construction of the complementation strain | |
| F-*waaF*C | TATGACCATGATTACGAATTCATGAAAATACTGGTGATCGGCC |
| R-*waaF*C | CAGGTCGACTCTAGAGGATCCTCAGGCTTCCTCTTGTAACAATAGC |
| F-*etp*C | CAGGTCGACTCTAGAGGATCCTTACCGGCTGAGGCGCTT |
| R-*etp*C | TATGACCATGATTACGAATTCATGGCCCAACTAAAATTTAACTCAA |
| F-*waaG*C | TATGACCATGATTACGAATTCATGATCGTTGCTTTTTGTTTATATAAATA |
| R-*waaG*C | CAGGTCGACTCTAGAGGATCCTCAACCATCCAGACCACCCG |
| F-*waaO*C | TATGACCATGATTACGAATTCATGTCTCAACTCAATGATAGTGACATCA |
| R-*waaO*C | CAGGTCGACTCTAGAGGATCCTTAGAAGCATTTTTCTTTATAATACTTTAAATAA |
| F-*waaR*C | TATGACCATGATTACGAATTCTTGGATTTTAAACATCTTACTCAATTTAAA |
| R-*waaR*C | CAGGTCGACTCTAGAGGATCCTTAACCTTTCATAACATTATATTTAATTGCT |
| F-*waaD*C | TATGACCATGATTACGAATTCATGGTTGATAAAATAATATTTACGGTTACTC |
| R-*waaD*C | CAGGTCGACTCTAGAGGATCCTTATTCAAACCAATTATGAATAACCTCTT |
| F-*waaL*C | TATGACCATGATTACGAATTCATGACCTCAACATTATTTTTCTCTCTCG |
| R-*waaL*C | CAGGTCGACTCTAGAGGATCCTTACTTGTTTTTCATCGCTAATAATAAGC |
| F-*ompC*C | TATGACCATGATTACGAATTCGATGAAAGTTAAAGTACTGTCCCTCCTG |
| R-*ompC*C | CAGGTCGACTCTAGAGGATCCTTAGAACTGGTAAACCAGACCCAGA |
| Tail fiber protein expression | |
| ORF165C-F | CAGCAAATGGGTCGCGGATCCAAGTTCAGGCAATACTTCTTG |
| ORF165C-R | GTGGTGGTGGTGGTGCTCGAGCCCGAAGGCCCTTTCTTATAA |
| ORF108C-F | CAGCAAATGGGTCGCGGATCCGGAGATAGTTGTTATTCATTGGGTACA |
| ORF108C-R | CAGCAAATGGGTCGCGGATCCGGAGATAGTTGTTATTCATTGGGTACA |
| ORF108C-GFP-F | CAGCAAATGGGTCGCGGATCCATGGTGAGCAAGGGCGAGG |
| ORF108C-GFP-R | ATAACAACTATCTCCGGATCCTTACTTGTACAGCTCGTCCATGCC |
| GFP-F | CAGCAAATGGGTCGCGGATCCATGGTGAGCAAGGGCGAGG |
| GFP-R | ACGGAGCTCGAATTCGGATCCTTACTTGTACAGCTCGTCCATGCC |
